# *N*^6^-methyladenosine modification of circNSUN2 facilitates cytoplasmic export and stabilizes *HMGA2* to promote colorectal liver metastasis

**DOI:** 10.1038/s41467-019-12651-2

**Published:** 2019-10-16

**Authors:** Ri-Xin Chen, Xin Chen, Liang-Ping Xia, Jia-Xing Zhang, Zhi-Zhong Pan, Xiao-Dan Ma, Kai Han, Jie-Wei Chen, Jean-Gabrie Judde, Olivier Deas, Feng Wang, Ning-Fang Ma, Xinyuan Guan, Jing-Ping Yun, Feng-Wei Wang, Rui-Hua Xu, Dan Xie

**Affiliations:** 1 State Key Laboratory of Oncology in South China; Collaborative Innovation Center for Cancer Medicine, Sun Yat-Sen University Cancer Center, Guangzhou, 510000 China; 2 Department of Oncology, the First Affiliated Hospital, Sun Yat-Sen University, Guangzhou, 510000 China; 3 Departments of Pathology, Sun Yat-Sen University Cancer Center, Guangzhou, 510000 China; 4 XenTech SAS, Evry, 91000 France; 5 Key Laboratory of Protein Modification and Degradation, School of Basic Medical Sciences, Affiliated Cancer Hospital & Institute of Guangzhou Medical University, Guangzhou, 510000 China; 6 Department of Clinical Oncology, the University of Hong Kong, Hong Kong, 999077 China

**Keywords:** Metastasis, Cell migration, Non-coding RNAs, RNA modification

## Abstract

Circular RNAs (circRNAs) have been implicated in cancer progression through largely unknown mechanisms. Herein, we identify an *N*^6^-methyladenosine (m^6^A) modified circRNA, circNSUN2, frequently upregulated in tumor tissues and serum samples from colorectal carcinoma (CRC) patients with liver metastasis (LM) and predicts poorer patient survival. The upregulated expression of circNSUN2 promotes LM in PDX metastasis models in vivo and accelerates cancer cells invasion in vitro. Importantly, *N*^6^-methyladenosine modification of circNSUN2 increases export to the cytoplasm. By forming a circNSUN2/IGF2BP2/*HMGA2* RNA-protein ternary complex in the cytoplasm, circNSUN2 enhances the stability of *HMGA2* mRNA to promote CRC metastasis progression. Clinically, the upregulated expressions of circNSUN2 and *HMGA2* are more prevalent in LM tissues than in primary CRC tissues. These findings elucidate that *N*^6^-methyladenosine modification of circNSUN2 modulates cytoplasmic export and stabilizes *HMGA2* to promote CRC LM, and suggest that circNSUN2 could represent a critical prognostic marker and/or therapeutic target for the disease.

## Abstract (web summary)

Liver metastasis of colorectal cancer leads to poor prognosis. Here the authors report that an *N*^6^-methyladenosine modified circular RNA is upregulated in colorectal cancer and promotes liver metastasis by enhancing the stability of *HMGA2* mRNA.

## Introduction

Colorectal carcinoma (CRC) ranks third in terms of incidence and remains the second leading cause of cancer-related mortality worldwide^1^. Clinically, metastasis is the overwhelming cause of death in patients with CRC, and the liver is the most frequent distant metastatic site. Approximately 15–25% of patients with CRC are diagnosed with liver metastasis (LM), which leads to poor prognosis beyond 5 years^2^. This poor long-term survival outlook highlights the need to understand the mechanisms of LM of CRC to further improve disease control. It is known that LM of CRC follows an ordered and hierarchical pattern, which comprises many steps: proliferation of the primary tumor, transfer to the circulating blood, adhesion to the liver sinusoids, and proliferation into the liver^3^. Although many previous reports have documented that an abundance of molecular abnormalities, in either coding proteins or noncoding RNAs, are involved and play important roles in the pathogenic process of LM of CRC^4–6^, the precise molecular mechanisms remain largely unclear.

*N*^6^-methyladenosine (m^6^A) was the first identified mammalian messenger RNAs (mRNAs) modification and remains the most abundant modification known in eukaryotic mRNAs and noncoding RNAs (ncRNAs)^7,8^. Circular RNAs (circRNAs) present a new class of small noncoding RNAs with a covalently single-stranded loop configuration that are produced from direct back-splicing or exon skipping of precursor mRNA (pre-mRNA)^9^. In the past two decades, circRNAs were considered to be byproducts of splicing errors with little functional potential^10^. Recently, with the improvement of RNA sequencing and computational approaches, a vast majority of circRNAs have been identified, and often exhibit tissue- or developmental stage-specific expression^9,11,12^. CircRNAs exhibit patterns of m^6^A modifications that are distinct from those of mRNAs^13^; however, the contributions of m^6^A modification for circRNAs metabolism have not been fully elucidated.

Dysregulated expressions of circRNAs have been identified in distinct pathological processes, including the pathogenesis of liver^14^, bladder^15^, lung^16^, and oral^17^ cancers. CircRNAs can function as miRNA sponges, modulating the activity of miRNAs on related target genes^14,15^ or regulate gene expression at both the transcription and splicing levels^18,19^; or can be translated, function as encoded protein^20,21^. These findings, taken together, suggest that circRNAs play functional roles in fundamental processes and serve as potential clinical molecular markers, thereby providing new insights into the treatment of human diseases, such as cancer. To date, however, the discovery of critical circRNA abnormalities, as well as their functions and/or underlying mechanisms, in the LM process of CRC, is still lacking.

In the present study, we demonstrate that a specific circRNA, circNSUN2, mapping to the chromosome 5p15 amplicon in CRC, is frequently upregulated in CRC patients with LM and predicts poorer disease survival. By using a patient-derived xenograft (PDX) CRC model, we find that higher expression of circNSUN2 potently promotes CRC LM. We further reveal that circNSUN2 is exported by YTH domain-containing protein 1 (YTHDC1) from the nucleus to the cytoplasm in an m^6^A methylation-dependent manner. Importantly, increased cytoplasmic circNSUN2 substantially enhances the stability of high mobility group AT-hook 2 (*HMGA2*) mRNA through interaction with an RNA-binding protein (RBP), Insulin-Like Growth Factor 2 mRNA-Binding Protein 2 (IGF2BP2), which consequently leads to the aggressive nature of CRC cells. Clinically, the upregulated circNSUN2 and *HMGA2* mRNAs are more prevalent in LM tissues than that in primary CRC tissues from the same patient. Our findings suggest that circNSUN2 functions as a critical predictor for LM of CRC patients and/or a potential therapeutic target against CRC.

## Results

### Profiling of deregulated circRNAs in CRC tissues

**Fig. 1:**
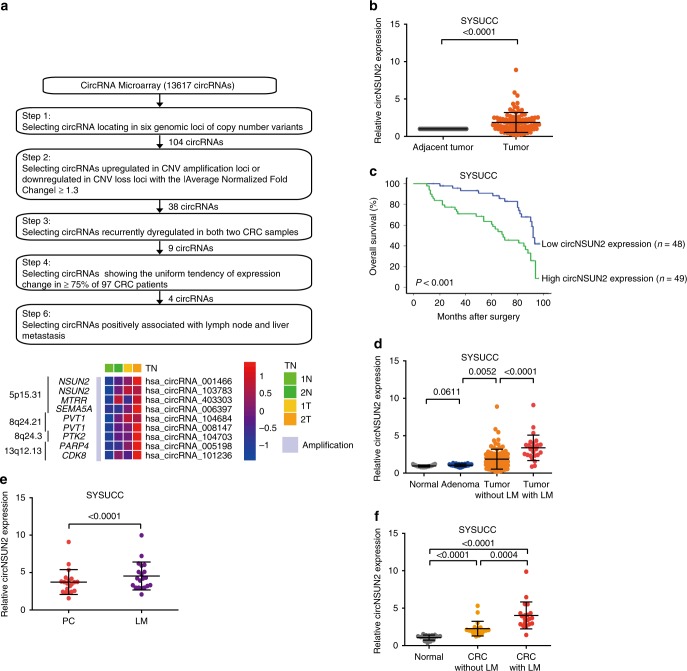
CircNSUN2 was upregulated in CRCs with liver metastasis (LM). **a** Top, flowchart illustrating the screening criteria of potential regulatory circRNAs enriched in CRCs. Bottom, clustered heatmap showing the dysregulated expression of circRNAs occurrent in both two CRC samples (the |average normalized fold change| ≥ 1.3) within susceptibility loci of CRC analyzed by CircRNAs Microarray. **b** qRT-PCR analysis of circNSUN2 expression in 97 CRC tissues and matched adjacent normal tissue. Data represent mean ± S.D., the *P* value was determined by a two-tailed paired Student’s *t* test. **c** Kaplan−Meier analysis of OS in CRC patients with low versus high expression the circNSUN2 from SYSUCC cohorts. The *P* value was determined by a Log-rank test. **d** qRT-PCR analysis of circNSUN2 expression from 18 normal colorectal tissues, 22 colorectal adenomas, 97 CRC patients without liver metastasis and 25 CRC patients with liver metastasis. Data represent mean ± S.D., the *P* values were determined by an unpaired Student’s *t* test. **e** qRT-PCR analysis of circNSUN2 expression in 20 pairs of primary colorectal cancer tissues (PC) and matched liver metastasis tissues (LM) surgically obtained from the same patients. Data represent mean ± S.D., the *P* value was determined by a two-tailed paired Student’s *t* test. **f** qRT-PCR analysis of circNSUN2 expression in serum from 18 normal control, 20 CRC patients without LM and 20 CRC patients with LM from the SYSUCC. Data represent mean ± S.D., the *P* values were determined by an unpaired Student’s *t* test

Genomic copy number aberrations are believed to be an important driver of tumorigenesis. In particular, copy number gains of 5p15.31, 8q24.21, 8q24.3 and 13q12.13, as well as losses of 5q21 and 18q21.1, have been identified as frequently occurring in CRCs^22–26^. To investigate the dynamics of circRNA alteration in the above genomic loci, we performed high-throughput CircRNA Microarray using two pairs of CRC and matched adjacent nontumor tissue samples. Within these genomic loci, we identified 38 dysregulated circRNAs meeting the following requirements: (1) upregulated in copy number variant (CNV) amplification loci or downregulated in CNV loss loci; (2) the |average normalized fold change| ≥ 1.3 (Supplementary Data 1). Among them, nine statistically significant and recurrently dysregulated circRNAs (occurrent in both two CRC samples) were further selected as validation candidates (Fig. 1a). Next, we compared the expression of nine circRNAs in tumor tissues compared to that in matched normal tissues derived from 97 CRC patients. While compared with the microarray data, we found that only four circRNAs showed the uniform tendency of expression change in ≥75% CRC patients (Fig. 1b and Supplementary Data1).

### The role of circNSUN2 in CRCs aggressiveness

To investigate the clinical significance among these dysregulated circRNAs in CRC patients, the cohort of 97 CRC patients with survival data was included (Supplementary Data 3). From Kaplan−Meier analyses, we found that high expression of circRNA_103783 (hg 19, chr5: 6623326–6625782), located on the chromosome 5p15.31 amplicon of CRC, suggested poorer patient overall survival (OS) (Fig. 1c). Notably, we found that high circRNA_103783 expression was positively associated with lymph node metastasis (Supplementary Tables 1 and 2). Utilizing the human reference genome (GRCh37/hg19), we noted that circRNA_103783 is derived from the exons 4 and 5 regions within the NOP2/Sun RNA methyltransferase family member 2 (NSUN2) locus; thus we termed it as circNSUN2.

Since lymph node metastasis is an important prognostic prediction factor in patients with LM^27,28^, which is the leading cause of CRC mortality^29,30^, we further investigate if circNSUN2 expression is associated with LM of CRC. We collected 25 cases of CRC with LM, as well as 18 cases of normal colorectal tissue and 22 cases of colorectal adenoma. We found that the levels of circNSUN2 were frequently upregulated in primary CRCs, particularly in CRCs with LM, when compared to the control groups of normal colorectal tissue and adenoma (Fig. 1d and Supplementary Table 3). Furthermore, we compared the expression of circNSUN2 in primary CRC (PC) and matched LM tissues surgically obtained from the same patients, and found a significant increase of circNSUN2 expression in LM tissues compared to paired PC tissues (Fig. 1e). Notably, circNSUN2 levels were significantly upregulated in serum derived from CRC patients with LM when compared to CRC patients without LM and normal controls (Fig. 1f).

### Characterization of circNSUN2 in CRCs

**Fig. 2:**
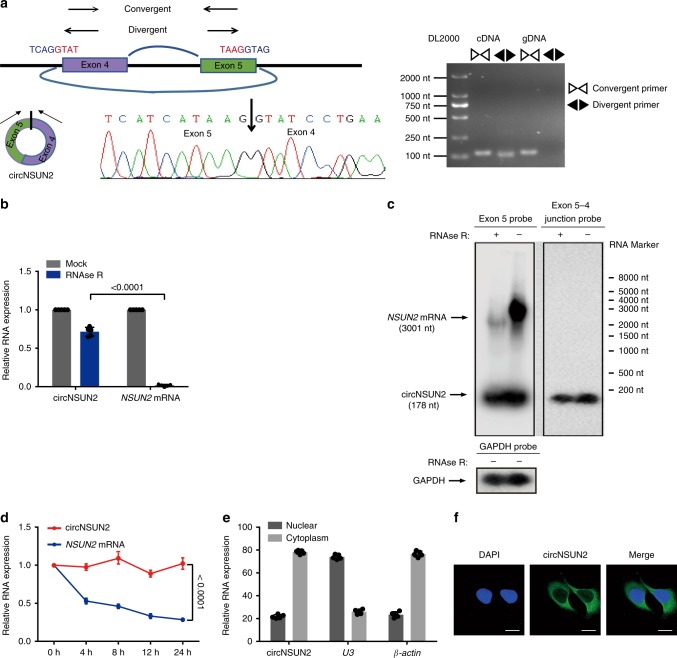
Characterization of circNSUN2 in CRC. **a** The genomic locus of circNSUN2. Left, the expression of circNSUN2 was detected by qRT-PCR followed by Sanger sequencing. Arrows represent divergent primers binding to the genome region of circNSUN2. Right, qRT-PCR products with divergent primers showing circularization of circNSUN2. cDNA complementary DNA. gDNA genomic DNA. **b** qRT–PCR analysis for the expression of circNSUN2 and *NSUN2* mRNA after treatment with RNase R in HCT116 cells. Data represent mean ± S.D. from five independent experiments; dot plot reflects data points from independent experiment. The *P* value was determined by a two-tailed unpaired Student’s *t* test. **c** Northern blotting of circNSUN2 and *NSUN2* transcripts by hybridization with exon5 (top, left) and exon 5−exon 4 junction (top, right) probes in the presence or absence of RNase R treatment. Right, RNA marker. *GAPDH* mRNA (bottom) was also blotted as an internal control. The samples for blotting *GAPDH* were aliquoted before RNase R treatment and loaded separately in two wells for validation. **d** qRT–PCR analysis for the expression of circNSUN2 and *NSUN2* mRNAs after treatment with Actinomycin D at the indicated time points in HCT116 cells. Data represent mean ± S.D. from five independent experiments; dot plot reflects data points from independent experiment. The *P* value was determined by a two-way ANOVA. **e** Cytoplasmic and Nuclear mRNA Fractionation experiment showing that circNSUN2 localized in the nucleus and the cytoplasm. *β-actin* and *U3* were applied as positive controls in the cytoplasm and nucleus, respectively. Data represent mean ± S.D. from five independent experiments; dot plot reflects data points from independent experiment. **f** RNA fluorescence in situ hybridization for circNSUN2. Nuclei were stained with DAPI. Scale bar, 10 µm. For **c** and **f**, junction probe is complementary to the junction sequence of circNSUN2. Source data are provided as a Source Data file

We compared circNSUN2 and *NSUN2* mRNA levels in a nontumorous tissue, three CRC tumor tissues, and five CRC cell lines by RT-PCR and real-time PCR (qRT-PCR). The expression levels of circRNA were quantified by qRT-PCR with divergent primers (Supplementary Data 2). CircNSUN2 was clearly upregulated in three CRC tumor tissues, five CRC cell lines compared to normal colorectal tissue, particularly in HCT116 cells (Supplementary Fig. 1a). Additionally, we compared the abundance of circNSUN2 with some other cancer-related circular RNAs, such as circHIPK3^31^ and circFOXO3 ^32,33^. Consistent with the expression levels in Circular Microarray, circNSUN2 was significantly more abundant compared to these cancer-related circular RNAs in HCT116 cell lines (Supplementary Fig. 1b). We aimed to characterize circNSUN2 by RT-PCR, Sanger sequencing (Fig. 2a), RNase R treatment (Fig. 2b), and northern blot (Fig. 2c) in HCT116 cells according to previously described methodology^18,33^. After examined by RT-PCR with divergent primers, the sequenced PCR product was corresponded to the 5′exon 5 to 3′exon 4 (Fig. 2a). Resistance to digestion with RNase R exonuclease confirmed that this RNA species harbors a circular RNA structure (Fig. 2b). We then used probes that hybridize with the exon 5−exon 4 junction to distinguish circNSUN2, and probes that hybridize with exon 5 to distinguish circNSUN2 and its host gene, *NSUN2*, by northern blotting (Fig. 2c). After treatment with Actinomycin D, an inhibitor of transcription, qRT-PCR analysis showed that the half-life of circNSUN2 exceeded 24 h, whereas that of the associated linear transcript exhibited about 4 h (Fig. 2d), indicating that circNSUN2 is more stable in CRC cells. Further nuclear and cytoplasmic fractionation (Fig. 2e) and fluorescence in situ hybridization (FISH) (Fig. 2f) examinations revealed that circNSUN2 was mainly localized in the cytoplasm but was also presented in the nucleus. These results, collectively, reveal that circNSUN2 is an abundant and stable circRNA expressed in CRC.

### CircNSUN2 promotes CRC cell metastasis in the PDX model

**Fig. 3:**
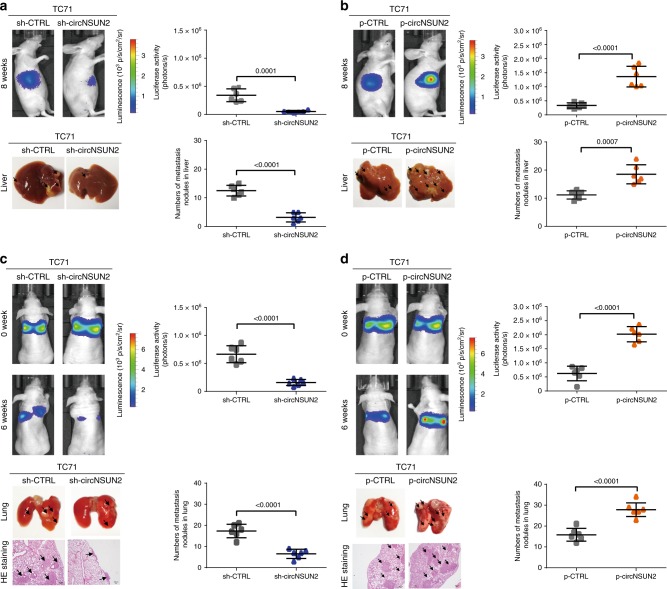
CircNSUN2 promotes metastasis of CRC. **a**, **b** Decreased (**a**) or increased (**b**) tumor metastasis formed in the livers of mice through the inferior hemispleen implantation of circNSUN2-konckdown (**a**) or circNSUN2-overexpression (**b**) TC71 PDX cells. Top, left, representative bioluminescent images of livers for each experimental group at 8 weeks. Top, right, statistical analysis of bioluminescent tracking plots. Bottom, left, representative liver. Bottom, right, the number of metastatic nodules formed in the lungs of mice for each group (*n* = 6 mice / group). Data represent mean ± S.D.; dot plot reflects data points from independent experiment. The *P* values were determined by a two-tailed unpaired Student’s *t* test. **c**, **d** Decreased (**c**) or increased (**d**) tumor metastasis formed in the lungs of mice through vein tail injection of circNSUN2-konckdown (**c**) or circNSUN2-overexpression (**d**) PDX cells. Top, left, representative bioluminescent images of lungs for each experimental group during 6 weeks. Top, right, statistical analysis of bioluminescent tracking plots. Bottom, left, representative lung and representative HE staining of lung metastatic lesions, Original magnification, ×4, scale bar, 100 µm. Bottom, right, the number of metastatic nodules formed in the lungs of mice for each group (*n* = 6 mice/group). Data represent mean ± S.D.; dot plot reflects data points from independent experiment. The *P* values were determined by a two-tailed unpaired Student’s *t* test. Source data are provided as a Source Data file

Given the important clinical relevance of circNSUN2 in CRC aggressiveness, we investigated the in vivo functions of circNSUN2 in CRC cell metastasis. To evaluate the biological functions of circNSUN2 in CRC, we first constructed circNSUN2 knockdown or overexpressed TC71 PDX CRC cells. The results demonstrated that after knockdown of circNSUN2 in PDX CRC cells, tumor metastasis was significantly inhibited compared to that of control cells in either liver (Fig. 3a) or lung (Fig. 3c) metastasis models. In contrast, nude mice injected with TC71 cells by overexpressing circNUSN2 had remarkably increased metastatic nodules in the liver (Fig. 3b) or lung (Fig. 3d) compared to controls. Additionally, the role of circNSUN2 in promoting metastasis of CRC was confirmed in a CRC cell line, HCT116 (Supplementary Fig. 2a−h). Additionally, we found that knockdown or overexpression of circNSUN2 had no effect on the expression of the host gene, *NSUN2* (Supplementary Fig. 1c, d), suggesting that the regulatory effect on CRC metastasis directly results from circNSUN2.

### CircNSUN2 enhances CRC cell aggressiveness in vitro

We performed a series of in vitro studies showing that knockdown of circNSUN2 significantly inhibited CRC cell migration (Supplementary Fig. 3a−c). Transwell (Supplementary Fig. 3a) and three-dimensional (3D) inverted invasion assays (Supplementary Fig. 3b) both revealed that depletion of circNSUN2 markedly reduced the invasion of CRC cells compared to control cells. By using 3D morphogenesis Matrigel cultures, we observed that knockdown of circNSUN2 in CRC cells resulted in attenuated invasion areas and numbers of protrusions (Supplementary Fig. 3c). In contrast, ectopic overexpression of circNSUN2 in CRC cells greatly increased cell migration and invasion (Supplementary Fig. 3d−f).

### YTHDC1 promotes cytoplasmic export of m^6^A modified circNSUN2

**Fig. 4:**
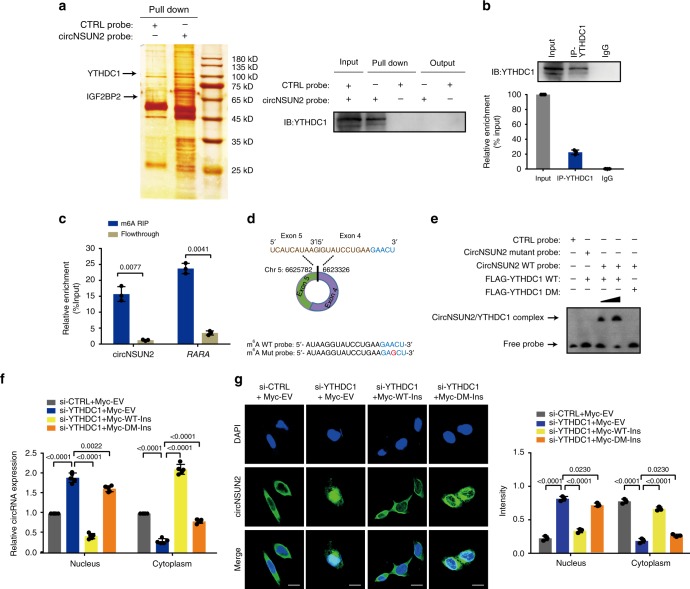
YTHDC1 promotes cytoplasmic export of m^6^A methylated circNSUN2. **a** Left, identification of the circNSUN2-protein complex pulled down by circNSUN2 junction probe with protein extracts from HCT116 cells. The arrows indicating the additional band presented in circNSUN2-protein complex. Right, immunoblot analysis of YTHDC1 after pulldown assay showing its specific association with circNSUN2. **b** RIP assays showing the association of YTHDC1 with circNSUN2. Top, IP efficiency of YTHDC1-antibody shown in western blotting. Bottom, relative enrichment representing RNA levels associated with YTHDC1 relative to an input control. IgG antibody served as a control. Data represent mean ± S.D. from three independent experiments; dot plot reflects data points from independent experiment. **c** MeRIP assay showing that circNSUN2 was highly recruited in m^6^A precipitated fraction. Data represent mean ± S.D. from three independent experiments; dot plot reflects data points from independent experiment. The *P* values were determined by a two-tailed paired Student’s *t* test. **d** Top, schematic illustration showing the GAACU m^6^A motif located at exon 5−exon 4 junction site of circNSUN2. Bottom, the sequence of RNA probe for RNA-EMSA assay. **e** RNA-EMSA assay showing the binding ability of purified YTHDC1 with biotin-labeled oligonucleotides containing GAACU motif from circNSUN2. **f** Cytoplasmic and Nuclear mRNA Fractionation experiment showing that knockdown of YTHDC1 increased the nuclear circNSUN2 content, whereas the dysregulation of nuclear circNSUN2 caused by YTHDC1 RNAi was recovered by overexpression of WT but not the mutant of YTHDC1. Data represent mean ± S.D. from five independent experiments; dot plot reflects data points from independent experiment. The *P* values were determined by a two-tailed unpaired Student’s *t* test. **g** RNA-FISH showing that the increased nuclear staining of circNSUN2 caused by YTHDC1 RNAi was rescued by overexpression of WT, but not the mutant YTHDC1. Scale bar, 10 µm. Data represent mean ± S.D. from three independent experiments; dot plot reflects data points from independent experiment. The *P* values were determined by a two-tailed unpaired Student’s *t* test. Source data are provided as a Source Data file. Unprocessed original scans of blots are shown in Supplementary Fig. 10

To explore the potential molecular mechanisms of circNSUN2 in regulating CRC malignance, we first performed RNA pull-down assays and mass spectrometry analysis to screen circNSUN2-interacting proteins (Fig. 4a and Supplementary Table 4). We verified YTHDC1 and IGF2BP2 as putative circNSUN2-binding proteins. Further RNA-binding protein immunoprecipitation (RIP) assays demonstrated the enrichment of circNSUN2 in complexes precipitated with antibody against YTHDC1 compared to those with control IgG (Fig. 4b).

To better map the interaction between YTHDC1 and circNSUN2, we applied a novel algorithm, circScan^34^, which is reliable to identify back-splicing junction reads in the human genomic location of circRNA from published RBP CLIP-seq data sets across various CLIP methods, including HITS-CLIP, PAR-CLIP, eCLIP, iCLIP and CLEAR-CLIP etc. From circScan^34^ analysis, we found that YTHDC1 and IGF2BP2 interacted with circNSUN2 at exon 5−exon 4 junction site of circNSUN2 (hg19, chr5: 6623326–6625782). Since YTHDC1 is known as an m^6^A reader^35^, we wondered if circNSUN2 contains m^6^A methylation. From methylated RNA immunoprecipitation (MeRIP) assays, we precipitated several known m^6^A-containing RNAs (such as *RARA*^36^, Fig. 4c). We found that exon 5−exon 4 junction sequence of circNSUN2 was also highly enriched in the m^6^A precipitated fraction (Fig. 4c) by using divergent primer of circNSUN2, confirming the m^6^A modification in circNSUN2. By browsing the exon 5−exon 4 junction sequence in circNSUN2, we identified that GAACU motif is a putative m^6^A motif (Fig. 4d, top). Consistently, we found that once mutated the GAACU m^6^A motif in RNA probe (Fig. 4d, bottom), the interaction ability of YTHDC1 at circNSUN2 was decreased from in vitro RNA electrophoretic mobility shift assays (RNA-EMSA) (Fig. 4e). Furthermore, once mutated the m^6^A-binding motif of YTHDC1, the interaction ability with circNSUN2 was decreased (Fig. 4e). These results indicated that YTHDC1 interacted with circNSUN2 with the m^6^A-binding motif at the GAACU m^6^A motif within the exon 5−exon 4 junction site of circNSUN2.

As the subcellular trafficking of circRNA remains largely unknown, given that YTHDC1 facilitates the nuclear export of m^6^A-modified mRNA^37^, we subsequently investigated if the export of m^6^A-modified circRNA relies on YTHDC1. Nuclear and cytoplasmic fractionation (Fig. 4f) and FISH (Fig. 4g) showed that silencing of YTHDC1 significantly increased the nuclear circRNA content. Enforced expression of YTHDC1 wild-type (WT), but not m^6^A-binding defective YTHDC1 (YTHDC1-DM)^35,38^, rescued the defective cytoplasmic export of circNSUN2 by depletion of YTHDC1 (Fig. 4f, g and Supplementary Fig. 4a). Taken together, these results provide evidence that YTHDC1 can bind to circNSUN2 and thus facilitate circNSUN2 export from the nucleus to the cytoplasm in an m^6^A-dependent manner.

In addition to that, we wondered if m^6^A methyltransferase METTL3 could affect circNSUN2 activity. We performed RNA-FISH assay, and found that silencing of METTL3 significantly increased the nuclear circRNA content. Enforced expression of METTL3 wild-type (WT), but not m^6^A-binding defective METTL3 (METTL3-BM) or m^6^A-catalytic defective METTL3 (METTL3-CM), restored the defective cytoplasmic export of circNSUN2 by METTL3 knockdown (Supplementary Fig. 4b, c).

We next investigated if m^6^A methylation of circNSUN2 plays an important role in CRC metastasis. We found that while circNSUN2 is overexpressed, the nuclear and cytoplasmic circNSUN2 contents were both increased, particularly in cytoplasmic fraction (Supplementary Fig. 4d). Moreover, m^6^A methylation levels were elevated (Supplementary Fig. 4e), and CRC cells invasion activity was promoted (Supplementary Fig. 4f−h). Once we mutated the GAACU m^6^A modification site in circNUSN2 overexpressing construct (Supplementary Fig. 8b), accompanied with the downregulated m^6^A modification level of circNUSN2 (Supplementary Fig. 4e), CRC cells invasion activity was attenuated (Supplementary Fig. 4f−h). Taken together, these results indicate that m^6^A modification of circNSUN2 is important for CRC cells invasion ability.

### CircNSUN2 interacts with IGF2BP2 through the CAUCAU motif

**Fig. 5:**
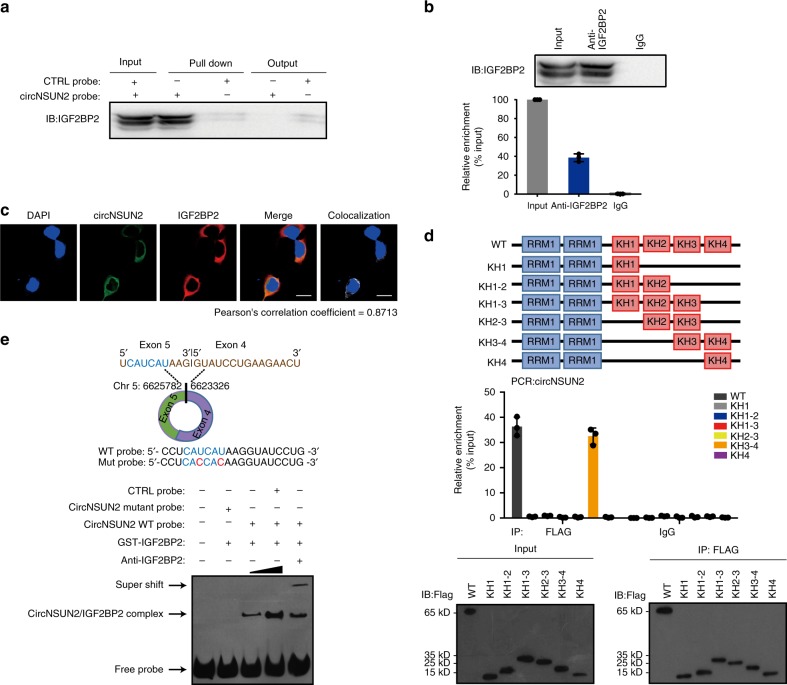
CircNSUN2 interacts with IGF2BP2 through CAUCAU motif. **a** Immunoblot analysis of IGF2BP2 after RNA-pulldown assay showing its specific association with circNSUN2. **b** RIP assays showing the association of IGF2BP2 with circNSUN2. Relative enrichment representing RNA levels associated with IGF2BP2 compared to an input control. IgG antibody served as a control. Data represent mean ± S.D. from three independent experiments; dot plot reflects data points from independent experiment. **c** IF-FISH assay showing that circNSUN2 is colocalized with IGF2BP2 protein in the cytoplasm. Scale bar, 10 µm. **d** Top, schematic structures showing RNA-binding domains within IGF2BP2 protein and a summary of IGF2BP2 truncations. Middle, relative enrichment representing circNSUN2 levels associated with truncated IGF2BP2 relative to an input control. Bottom, immunoblot analysis with anti-FLAG of HCT116 cells transfected with plasmids encoding FLAG-tagged WT or truncated IGF2BP2s. Data represent mean ± S.D. from three independent experiments; dot plot reflects data points from independent experiment. **e** Top, schematic illustration showing the CAUCAU motif located at exon 5−exon 4 junction site of circNSUN2 and the RNA probe for RNA-EMSA assay. Bottom, RNA-EMSA assay showing the binding ability of purified IGF2BP2 with biotin-labeled oligonucleotides containing CAUCAU motif from circNSUN2. Source data are provided as a Source Data file. Unprocessed original scans of blots are shown in Supplementary Fig. 10

From RNA pull-down assays, we first observed that circNSUN2 was pull-downed with abundant IGF2BP2 protein (Figs. 4a, 5a and Supplementary Table 4). Further RNA immunoprecipitation confirmed the interaction between IGF2BP2 and circNSUN2 (Fig. 5b). By performing immunofluorescence and fluorescence in situ hybridization (IF-FISH) assays, we confirmed the colocalization of endogenously expressed circNSUN2 and IGF2BP2 in the cytoplasm (Fig. 5c). These results suggest that circNSUN2/IGF2BP2 form an RNA-protein complex in the cytoplasm.

Next, we studied which domain of IGF2BP2 contributes to the interaction with circNSUN2. We constructed IGF2BP2 mutants with truncation of individual KH domains. Further RIP assays (Fig. 5d) revealed that the KH3-4 di-domain of IGF2BP2 specifically bound to circNSUN2, suggesting that the KH3-4 di-domain is responsible for recruiting circNSUN2. On the other side, we searched for the motif within circNSUN2 that is indispensable for IGF2BP2 recruitment. To better map the interaction between IGF2BP2 and circNSUN2, we applied circScan^34^ analysis, we found that IGF2BP2 bound to circNSUN2 at exon 5−exon 4 junction site of circNSUN2 (hg19, chr5: 6623326–6625782). It was shown by Markus et al. that the sequence CAUH (H = A, U or C) as the only consensus recognition element for IGF2BP2 ^39^. Therefore, by browsing the exon 5−exon 4 junction sequence in circNSUN2, we identified that CAUCAU motif located at the exon 5−exon 4 junction is a putative binding motif of IGF2BP2 (Fig. 5e, top). By in vitro RNA-EMSA, we validated that the CAUCAU motif inside of circNSUN2 is required for IGF2BP2 interaction. Super shift experiments indicated that IGF2BP2 specifically binds to this sequence. When the concentration of IGF2BP2 protein was increased, the concentration of the circNSUN2/IGF2BP2 mixture was also increased. Mutation of the CAUCAU motif significantly reduced the concentration of the mixture (Fig. 5e, bottom). Our data collectively reveal that IGF2BP2 binds to the CAUCAU motif of circNSUN2 through the KH3-4 di-domain.

### CircNSUN2/IGF2BP2/*HMGA2* complex stabilizes *HMGA2* mRNA

As IGF2BP2 is essential for mRNA stability^40,41^, we then wondered if the circNSUN2/IGF2BP2 complex stabilizes certain unknown downstream targets. Therefore, we performed RNA-SEQ analyses in HCT116 cells (Supplementary Fig. 5a); 644 mRNAs showed a significant decrease in mRNA expression upon circNSUN2 silencing (fold change > 1.5). It has been reported that IGF2BP2 preferentially binds to 3′UTR of target mRNAs with high AU content^39,40^. Therefore, among the 644 downregulated mRNAs, we screened IGF2BP2-binding 3′UTRs from published RBP CLIP-SEQ data sets across various cancer types, including Starbase^42^ and IGF2BP2 Enhanced-CLIP SEQ data^43^. After screening, we identified 21 mRNAs bound by IGF2BP2 (Supplementary Table 5). Given that circNSUN2 promotes the metastasis progression, therefore, based on the results reported in the literatures, ten metastasis-related genes were identified as potential targets of circNSUN2 (Supplementary Table 5). Through further qRT-PCR and WB validation, we confirmed that *HMGA2* is the target of circNSUN2. By using sequence BLAST analysis, we found that the AAACA site inside of circNSUN2 can directly bind to the 3′UTR of *HMGA2* with AU-Rich Elements. Therefore, we selected *HMGA2* for further investigation.

**Fig. 6:**
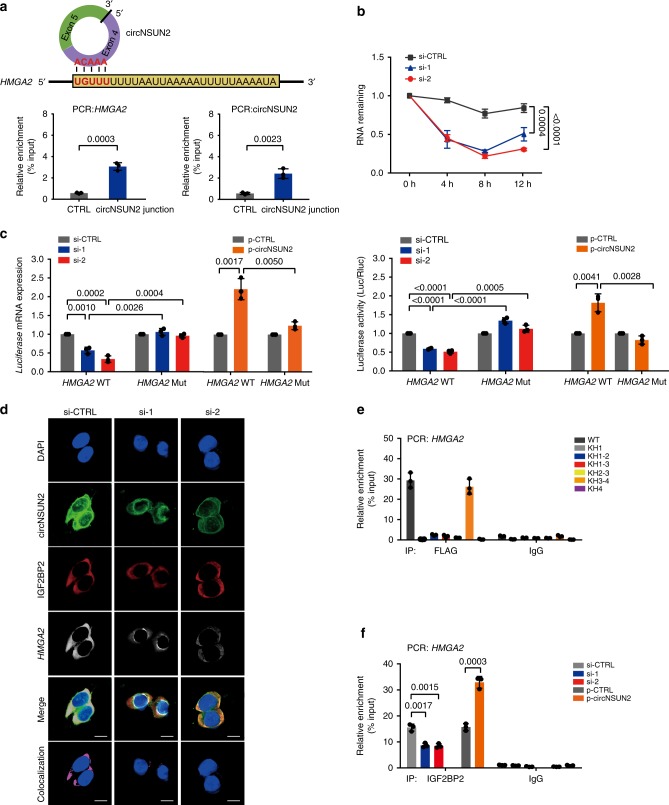
CircNSUN2/IGF2BP2/*HMGA2* ternary complex stabilized *HMGA2* mRNA. **a** Top, sequence BLAST analysis showing that circNSUN2 directly targets the 3′UTR of *HMGA2* with high AU content. Bottom, relative enrichment representing *HMGA2* (left panel) and circNSUN2 (right panel) RNA levels associated with circNSUN2 junction compared to control. Data represent mean ± S.D. from three independent experiments; dot plot reflects data points from independent experiment. The *P* values were determined by a two-tailed unpaired Student’s *t* test. **b** CircNSUN2 knockdown in HCT116 cells significantly downregulated *HMGA2* mRNA abundance. Data represent mean ± S.D. from three independent experiments; dot plot reflects data points from independent experiment. The *P* values were determined by a two-way ANOVA. **c** Left, *luciferase* mRNA expression of luciferase reporter gene with *HMGA2*-WT or *HMGA2*-Mut in control and circNSUN2-knockdown or circNSUN2-overexpression HCT116 cells. Right, relative luciferase activity of luciferase reporter gene with *HMGA2*-WT or *HMGA2*-Mut in control and circNSUN2-knockdown or circNSUN2-overexpression HCT116 cells. Data represent mean ± S.D. from three independent experiments; dot plot reflects data points from independent experiment. The *P* values were determined by a two-tailed unpaired Student’s *t* test. **d** IF-FISH assay showing that the colocalization of circNSUN2/IGF2BP2/*HMGA2* was decreased upon knockdown of circNSUN2. Scale bar, 10 µm. **e** Relative enrichment representing the enrichment of *HMGA2* associated with truncated IGF2BP2 protein complex compared to an input control. IgG antibody served as a control. Data represent mean ± S.D. from three independent experiments; dot plot reflects data points from independent experiment. **f** RIP assays showing the association of IGF2BP2 with *HMGA2* upon circNSUN2 silencing or overexpression. Data represent mean ± S.D. from three independent experiments; dot plot reflects data points from independent experiment. The *P* values were determined by a two-tailed unpaired Student’s *t* test. Source data are provided as a Source Data file

The interaction between circNSUN2 and *HMGA2* was confirmed by RNA pull-down assays (Fig. 6a). We further found that knockdown of circNSUN2 significantly reduced the mRNA stability of *HMGA2* (Fig. 6b), which consequently caused the reduction of HMGA2 expression (Supplementary Fig. 5b). Consistently, the expression of C-X-C motif chemokine receptor 4 (CXCR4), a downstream target of HMGA2^44^, was also downregulated upon circNSUN2 depletion (Supplementary Fig. 5b). Conversely, enforced expression of circNSUN2 efficiently upregulated the expression of both HMGA2 and CXCR4 (Supplementary Fig. 5c).

As reported by Li et al., HMGA2 induces epithelial-to-mesenchymal transition (EMT) and contributes to colon cancer progression in colon cancer^45^; we further assessed if circNSUN2 could enhance CRC cells EMT phenotype. Western blot (WB) showed that overexpression of circNSUN2 led to the downregulated expression of the epithelial marker E-cadherin, and upregulated expression of the mesenchymal marker, Vimentin, in CRC cells (Supplementary Fig. 5c), suggesting that circNSUN2 promotes EMT in CRC cells through HMGA2 pathway.

To further investigate whether the formation of the circNSUN2/*HMGA2* complex is indispensable for *HMGA2* mRNA stabilization, we constructed luciferase reporter minigenes containing wild-type *HMGA*2-3′UTR (*HMGA2*-WT) or mutant 3′UTR (*HMGA2*-Mut), respectively. For the mutant form of *HMGA2*-3′UTR luciferase reporter, the TGTTT motif, which is required for the interaction with circNSUN2, was replaced. The luciferase activity of *HMGA2*-Mut was half less than that of control *HMGA2*-WT (Supplementary Fig. 5d). In addition to that, knockdown of circNSUN2 dramatically inhibited the *luciferase* mRNA expression (Fig. 6c, left) and luciferase activity (Fig. 6c, right) of *HMGA2*-WT, but not that of *HMGA2*-Mut. Conversely, overexpression of circNSUN2 dramatically increased the *luciferase* mRNA expression (Fig. 6c, left) and luciferase activity (Fig. 6c, right) of *HMGA2*-WT, but not that of *HMGA2*-Mut.

We further revealed that circNSUN2/*HMGA2*/IGF2BP2 formed an RNA-protein ternary complex; this was supported by the following two lines of evidence. First, RNA-FISH assays showed that *HMGA2* and IGF2BP2 are colocalized in the cytoplasm. In the absence of circNSUN2, the colocalization of the *HMGA2*/IGF2BP2 RNA−protein complex was significantly decreased (Fig. 6d and Supplementary Fig. 5e) while IGF2BP2 expression was unaltered. Second, our RIP assays showed that the KH3-4 di-domain of IGF2BP2 was required for its interaction with circNSUN2 and *HMGA2* (Figs. 5d, 6e). Knockdown of circNSUN2 markedly reduced the *HMGA2*/IGF2BP2 RNA−protein interaction as shown in the RIP assays, whereas ectopic overexpression of circNSUN2 significantly increased the enrichment of *HMGA2* in IGF2BP2 immunoprecipitated fractions (Fig. 6f and Supplementary Fig. 5f). These findings demonstrate that circNSUN2 plays a critical role in promoting the interactions between IGF2BP2 and *HMGA2*, and enhances the mRNA stability of *HMGA2* through the formation of a circNSUN2/IGF2BP2/*HMGA2* RNA−protein ternary complex.

### CircNSUN2 promotes LM of CRC through the HMGA2 pathway

**Fig. 7:**
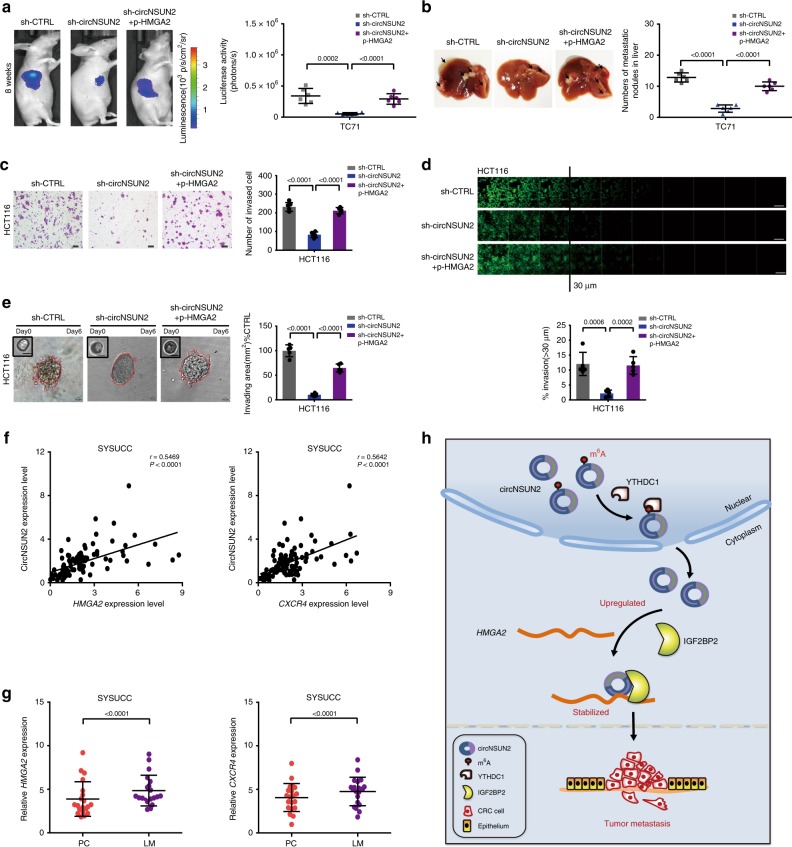
CircNSUN2 promotes LM metastasis of CRC through HMGA2 pathway. **a**, **b** Decreased tumor metastasis formed in the livers of mice through the inferior hemispleen implantation of circNSUN2-konckdown TC71 PDX cells was rescued by overexpression of HMGA2. **a** Left, representative bioluminescent images of livers for each experimental group at 8 weeks. Right, statistical analysis of bioluminescent tracking plots. The *P* values were determined by a two-tailed unpaired Student’s *t* test. **b** Left, representative liver. Right, the number of metastatic nodules formed in the livers of mice for each group (*n* = 6 mice/group). The *P* values were determined by a two-tailed unpaired Student’s *t* test. **c**−**e** Transwell assay (**c**), inverted invasion assay (**d**) and 3D multicellular tumor spheroids invasion assay (**e**) showing that decreased cell invasion in circNSUN2 knockdown HCT116 cells was rescued by overexpression of HMGA2. Scale bar, 100 µm (**c**), 100 µm (**d**) and 10 µm (**e**). Data represent mean ± S.D. from five independent experiments; dot plot reflects data points from independent experiment. The *P* values were determined by a two-tailed unpaired Student’s *t* test. **f** Left, circNSUN2 expression showing positively correlated with *HMGA2* expression in CRC patients derived from SYSUCC. Right, circNSUN2 expression showing positively correlated with *CXCR4* expression in CRC patients derived from SYSUCC. The *P* values were determined by Pearson correlation analysis. **g** qRT–PCR analysis for the RNA expression of *HMGA2* and *CXCR4* in 20 pairs of primary colorectal cancer (PC) and matched liver metastasis (LM) surgically obtained from the same patients. Data represent mean ± S.D., the *P* values were determined by a two-tailed paired Student’s *t* test. **h** A proposed model for the regulatory landscape of the circNSUN2/IGF2BP2/*HMGA2* signaling axis in promoting the metastasis of CRC. Source data are provided as a Source Data file

We then investigated if the role of circNSUN2 in metastasis of CRC is dependent on the HMGA2 pathway. In vivo model showed that the decreased metastatic nodules in the liver formed by injection with the circNSUN2-knockdown PDX CRC cells were largely restored by overexpression of HMGA2 (Fig. 7a, b). In vitro assays by Transwell assays (Fig. 7c), 3D inverted invasion assays (Fig. 7d) and 3D morphogenesis Matrigel cultures (Fig. 7e) further demonstrated that enforced expression of HMGA2 functionally rescued the decreased cell invasion and migration upon circNSUN2 silencing. In addition, overexpression of the circNSUN2 mutant (Supplementary Fig. 8c) that lacks *HMGA2* binding ability substantially led to reduced migration and invasion in CRC cells (Supplementary Fig. 6a−c).

We next compared the in vivo expression levels of *HMGA2* and *CXCR4* between liver metastatic nodules in mice injected with control or circNSUN2-silenced PDX CRC cells. We found that downregulations of *HMGA2* and *CXCR4* in liver metastatic nodules were consistent with that of circNSUN2 silencing (Supplementary Fig. 7a, b). Conversely, the upregulations of *HMGA2* and *CXCR4* in liver metastatic nodules were consistent with that of circNSUN2 overexpression (Supplementary Fig. 7c, d). These data suggest that the oncogenic functions of circNSUN2 in promoting CRC cell metastasis rely on the HMGA2 pathway.

### CircNSUN2/*HMGA2*/*CXCR4* is positively associated with CRC LM

To further reveal the clinical relevance of circNSUN2 regulation in CRC, we examined the expression levels of circNSUN2, *HMGA2* and *CXCR4* in a cohort of 97 CRC patients. We found that the expression levels of *HMGA2* and *CXCR4* were positively correlated with the levels of circNSUN2 transcripts (Fig.7f). Notably, the expression levels of *HMGA2* and *CXCR4* predicted poorer CRC patient OS (Supplementary Fig. 7e, f).

We next examined the expression levels of *HMGA2* and *CXCR4* in PC and matched LM tissues surgically obtained from the same 20 CRC patients. The results showed that upregulated expressions of *HMGA2* and *CXCR4* were more prevalent in LMs than in PCs (Fig. 7g).

## Discussion

In this study, we first demonstrated that circNSUN2, which maps to the 5p15 amplicon in CRCs, is an important circRNA that promotes LM in CRC. We showed that circNSUN2 is frequently upregulated in CRC patients with LM and predicts poorer patient survival. By using a PDX model, we found that higher expression of circNSUN2 promotes LM in CRCs. We revealed that the nuclear export of circNSUN2 is mediated by YTHDC1 in an m^6^A methylation-dependent manner. Importantly, increased cytoplasmic expression of circNSUN2 enhances the stability of *HMGA2* mRNA by forming a circNSUN2/IGF2BP2/*HMGA2* RNA−protein ternary complex, which consequently leads to the LM of CRC. Clinically, the expressions of circNSUN2, *HMGA2* and *CXCR4* are significantly associated with advanced T status and occur more frequently in LM compared to PCs.

While m^6^A is recognized as an abundant cotranscriptional modification in mRNAs and ncRNAs^7,8^, including circRNAs^13^, and is implicated in numerous aspects of post-transcriptional mRNA metabolism^38,46,47^, little is known about the effects of m^6^A modification on cellular circRNAs biology. The biogenesis of circRNAs has been widely studied from different aspects. Generally, circRNAs are regarded as cotranscriptional products resulting from canonical linear mRNA splicing, which occurs in the nucleus^9^. However, the majority of circRNAs have been found localized in the cytoplasm. Therefore, it is critical to investigate the underlying mechanisms that regulate the export of circRNAs from the nucleus to the cytoplasm. Recent findings have demonstrated that *Drosophila* Hel25E and its human homologs, UAP56/URH49, regulate circRNAs localization and control the efficiency of nuclear export by measuring the lengths of mature circRNAs^48^. Our study provides the first evidence that the export of circNSUN2 from the nucleus to the cytoplasm is dependent on m^6^A modification and is mediated through the recruitment of YTHDC1. Moreover, although m^6^A modification has been reported as widespread in circRNAs and can be recognized by YTHDF1 and YTHDF2, m^6^A-modified circRNAs exhibit less stability when regulated by YTHDF2^13^. On the other hand, YTHDC1 is shown to interact with splicing factors SRSF1, SRSF3, SRSF7, SRSF7 and SRSF10, regulating the splicing of mRNA^38^. Additionally, YTHDC1 facilitates mRNA binding to both SRSF3 and the canonical export receptor NXF1, mediating export and metabolism of m^6^A-modified mRNAs^37^. We identified that circNSUN2 is interacted with YTHDC1, SRSF3 and NXF1 from mass spectrometry (Fig. 4a, PeptideAtlas Dataset ID: PASS01424), suggesting that the export of circNSUN2 from the nucleus is through YTHDC1-dependent export. Herein, our results strongly suggest that YTHDC1 exhibits a distinct function compared with YTHDF2 in promoting the cytoplasmic export of m^6^A-modified circNSUN2 and supporting an emerging paradigm of m^6^A as a potential selective signal for the metabolism of mammalian circular RNAs.

So far, there are three well-studied mechanisms by which circRNAs exert their biological functions. The first is that nuclear retained circular RNAs can regulate gene expression at both the transcription and splicing levels^14,15^. The second is that circRNAs can be translated and function as encoded proteins^18,19^. The third, which has been widely demonstrated, is that circRNAs can act as sponges for miRNAs through their binding sites to modulate the activity of miRNAs on other target genes^20,21^. Herein, we provide a potent mechanism by which circNSUN2 can enhance the stability of *HMGA2* mRNA by forming a circNSUN2/IGF2BP2/*HMGA2* RNA-protein ternary complex. Therefore, from the aspect of circRNA regulation, we discovered a distinctive function by which circRNAs can enhance mRNA stabilization through interacting with RBPs. Interestingly, for IGF2BP2, we identified a regulatory mechanism of mRNA stabilization in which IGF2BP2 enhances the stabilization of *HMGA2* in an m^6^A modification-independent manner, providing an important role for circRNAs in RNA metabolism.

Genomic copy number aberrations are believed to be the driver of tumor carcinogenesis through amplification of oncogenes, inactivation of tumor suppressor genes, or more subtle gene dosage changes. In particular, copy number gains of 5p15.31, 8q24.21, 8q24.3 and 13q12.13, as well as losses of 5q21 and 18q21.1, have been identified as frequently occurring in a substantial fraction of CRC tissues^22–26^. To date, studies of these CRC susceptibility loci have mainly focused on a limited number of encoding genes^22,49–52^. Thus, to further reveal the potential regulatory mechanisms under these genomic loci, studies on the pathological functions of small noncoding RNAs are required. CircRNAs are a novel type of noncoding small RNAs that form a covalently closed loop structure^9^. During the past 20 years, a large number of exonic and intronic circRNAs have been identified among eukaryotes,^11,53^ indicating that circRNAs are not simply aberrant splicing byproducts but rather have multiple potential biological functions. Our study identified that circNSUN2, arising from the 5p15.31 amplicon, is frequently upregulated in CRCs with LM and is related to poorer patient OS.

Although dysregulation of circRNAs has been reported in CRC tissues previously^54^, no prior study has investigated their functions in LM of CRC or explored their feasibility as invasive biomarkers in serum of CRC patients. This is the first study to explore the clinical relevance of circNSUN2 expression in CRC patients with LM and matched serum specimens. We observed a significant increase of circNSUN2 expression in tumor tissues and matched serum from CRC patients with LM. Our data from clinical specimens implies that circNSUN2 may serve as a diagnostic and/or prognostic marker of CRC patients with LM.

Another unique strength of our study is that we were able to substantiate our results by using human clinical CRC specimens implanted into an in vivo metastasis PDX model. We identified that high expression of circNSUN2 in CRC cells dramatically promoted LM of CRC in the PDX model. We recognize that although our liver and/or systemic metastasis mouse PDX models may not reproduce the complexity of metastatic CRC in human beings, our results from this animal model strongly support the clinical data and in vitro functions of circNSUN2 in promoting LM of CRC.

In conclusion, we provide the first line of comprehensive evidence that circNSUN2 is an important oncogenic circRNA as well as a diagnostic/prognostic biomarker for CRCs with LM. CircNSUN2 exerts a critical role in stabilizing *HMGA2* mRNA by forming a circNSUN2/IGF2BP2/*HMGA2* ternary complex to promote CRC cell aggressiveness (Fig. 7h). Importantly, our findings may offer a potential therapeutic target, circNSUN2, to broaden the treatment options for human CRC, especially the disease with LM.

## Methods

### Patients and tissue specimen collection

This study has been approved by Institutional Review Board of Sun Yat-Sen University Cancer Center (SYSUCC, Guangzhou, China), and the study was informed in accordance with Declaration of Helsinki. Written informed consent was obtained from the patients before the study began.

Human CRC and adjacent normal tissues were collected from 97 patients receiving surgery with informed consent at Sun Yat-Sen University Cancer Center (SYSUCC) from 2005 to 2015. Clinical information of the CRC patients is summarized in Supplementary Data 3. The tumor grade and stage were defined according to the criteria of the World Health Organization (WHO) and the sixth edition of the TNM classification of the International Union Against Cancer (UICC, 2009). All of the patients were followed up on a regular basis, overall survival (OS) time was determined from the date of surgery to the date of death or the date of the last follow-up visit for survivors.

A total of 18 normal colorectal tissues and 22 colorectal adenomas were collected. In addition, 25 CRC tissues (including 20 pairs with matched LM) were obtained from SYSUCC. Clinical information of the CRC patients is summarized in Supplementary Data 4. Serum samples from 18 healthy individuals, 20 CRC patients without LM and 20 CRC patients with LM were used in this study.

### Cell cultures

The DLD1 (ATCC CCL-221), HCT116 (ATCC CCL-247), LoVo (ATCC CCL-229), SW620 (ATCC CCL-227), SW1116 (ATCC CCL-233) and 293T (ATCC CRL-11268) cell lines were purchased from American Type Culture Collection (ATCC). The TC71 patient-derived xenograft cell line was obtained from XENTECH (MRF reference: XTM-233_CXT-399/R5700).

DLD1, HCT116, LoVo, SW620 and SW1116 were cultured in RPMI-1640 medium (Invitrogen, Carlsbad, USA) with 10% fetal bovine serum (HyClone, USA) as routine. 293T was maintained in Dulbecco’s modified Eagle’s medium (Invitrogen, Carlsbad, USA) supplemented with 10% fetal bovine serum. TC71 was propagated in Advanced DMEM/F12 supplemented with 8% FBS, 1% antibiotics and 1% Glutamin. All cells were grown in a humidified incubator at 37 °C with 5% CO_2_. All the cell lines were authenticated 3 months before the beginning of the study based on viability and morphology by the suppliers. Cells have not been in culture for longer than 2 months.

### Microarray analysis

Samples (two CRC and paired adjacent normal tissues) were obtained from surgical specimens at the Cancer Center. The sample preparation and microarray hybridization were performed according to the Arraystar’s standard protocols (Rockville, MD, USA). Circular RNAs were amplified by digestion with RNase R (Epicentre Technologies, Madison, WI, USA) to remove linear RNAs and transcribed into fluorescent circRNA by the use of Arraystar Super RNA Labeling Kit (Arraystar). Subsequently, the labeled circRNAs were hybridized onto the Arraystar Human circRNA Array V2 (8 × 15 K, Arraystar), and then scanned by the Agilent Scanner G2505C (Jamul, CA, USA). Differentially expressed circRNAs demonstrating statistical significance (the |average normalized fold change| ≥ 1.3) between groups were identified by utilizing fold change cut-off, respectively.

### RNA interference (RNAi) and transfection

shRNAs for knockdown of circNSUN2 were obtained from GeneCopoeia (MD, USA). siRNAs duplexes were synthesized by GenePharma (Suzhou, China). The target sequences for constructing lentiviral shRNAs and siRNAs are listed in Supplementary Data 2.

### Plasmid construction

CircNSUN2 overexpression plasmid, circNSUN2-m^6^A mutation plasmid and circNSUN2-*HMGA2* binding mutation plasmid were obtained from Furuibio (Guangzhou, China). Flag-IGF2BP2 WT and truncated plasmids, HMGA2 overexpression plasmid were obtained from VigeneBio (Maryland, USA). SFB-YTHDC1-WT, SFB-YTHDC1-DM, Myc-YTHDC1-WT Ins, Myc-YTHDC1-DM Ins, Myc-METTL3-WT Ins, Myc-METTL3-BM Ins, and Myc-METTL3-CM Ins constructs were obtained from Dr. Yun-Gui Yang (Beijing Institute of Genomics, Chinese Academy of Sciences, Beijing, China).

Plasmid transfection was performed with Lipofectamine 3000 (Invitrogen, CA, USA) according to the manufacturers’ instructions. For stable transductions, lentivirus production and infection were performed with Lenti-Pac HIV package kit and concentrated with Concentration of lentiviral particles (GeneCopoeia, MD, USA) according to the manufacturers’ instructions. TC71 and HCT116 cells were transduced with lentiviral vectors containing Gaussia luciferase (Gluc) for in vivo bioluminescence imaging. Puromycin or Geneticin was used for several days to select stable cells^55^.

### Antibodies

The antibodies were purchased from commercial sources:

anti-YTHDC1 for western blot (1:1000 dilutions, ab122340, Abcam, Cambridge, UK); anti-METTL3 for western blot (1:1000 dilutions, ab195352, Abcam, Cambridge, UK); anti-IGF2BP2 for western blot (1:1000 dilutions, 11601-1-AP, Proteintech, Chicago, USA); anti-Flag for western blot (1:1000 dilutions, 8146, Cell Signaling, Boston, USA); anti-Myc for western blot (1:1000 dilutions, 2276, Cell Signaling, Boston, USA); anti-NSUN2 for western blot (1:1000 dilutions, 66580-1-Ig, Proteintech, Chicago, USA); anti-HMGA2 for western blot (1:500 dilutions, 20795-1-AP, Proteintech, Chicago, USA); anti-CXCR4 for western blot (1:1000 dilutions, 11073-1-AP, Proteintech, Chicago, USA); anti-E-cadherin for western blot (1:1000 dilutions, 610405, BD Biosciences, New Jersey, USA); anti-Vimentin for western blot (1:1000 dilutions, 550513, BD Biosciences, New Jersey, USA); anti-GAPDH for western blot (1:5000 dilutions, 60004-1-Ig, 10494-1-AP, Proteintech, Chicago, USA); anti-IGF2BP2 for immunofluorescence (1:200 dilutions, 11601-1-AP, Proteintech, Chicago, USA); Key Fluor 647-conjugated goat anti-rabbit IgG (H + L) for immunofluorescence (1:200 dilutions, KGAB015, Keygen, Nanjing, China); anti-m6A for MeRIP (10 μg antibody for 5 μg mRNAs, 202003, Synaptic Systems, Goettingen, Germany); anti-IGF2BP2 for RIP (1:100 dilutions, 11601-1-AP, Proteintech, Chicago, USA); anti-Flag for RIP (1:100 dilutions, 8146, Cell Signaling, Boston, USA); anti-Myc for RIP (1:100 dilutions, 2276, Cell Signaling, Boston, USA); anti-IgG for RIP (1:100 dilutions, CS200621, PP64B, Millipore, Massachusetts, USA)

### RNA quantitative real-time polymerase chain reaction

Total RNA was extracted using TRIzol reagent (Invitrogen, Carlsbad, USA). Two micrograms of total RNAs was used to synthesize cDNA, a portion of which (1 µl, equal to 0.2 µg cDNA) were used in a PCR. Real-time polymerase chain reaction (RT-PCR) was carried out using SYBR Green SuperMix (Roche, Basel, Switzerland) and ABI7900HT Fast Real-Time PCR system (Applied Biosystems, CA, USA) using 1 µl cDNA as template^32,33^. Either *β-actin* or *U3* was used as an internal control. The primer sequences are listed in Supplementary Data 2.

### Western blotting

Equal amounts of protein lysates were resolved by SDS-PAGE gels and then transferred on a PVDF membrane (Millipore, Massachusetts, USA). After incubation with a primary antibody at 4 °C overnight, the membranes were hybridized with a secondary antibody at room temperature for 1 h. The immunoreactive signals were visualized by enhanced chemiluminescence kit (Amersham Biosciences, Uppsala, Sweden).

### Actinomycin D and RNase R treatment

HCT116 cells were planted into six-well plates. Up to 60% confluency after 24 h, cells were treated with 5 μg/ml Actinomycin D or DMSO and collected at indicated time points.

Total RNA (2 μg) was incubated with 3 U/μg of RNase R (Epicentre Technologies, Madison, WI, USA) for 15 min at 37 °C. After treatment with Actinomycin D or RNase R, the RNA expression levels of circNSUN2 and other mRNAs were analyzed by qRT-PCR.

### Northern blotting

The labeled RNA probes were synthesized from Sangon Biotech (Shanghai, China). Northern blotting was performed using NorthernMax Kit from Ambion (Life Technologies, Carlsbad, USA). Ten micrograms total RNA with RNase R digestion was loaded on a 2% agarose gel and transferred to a Hybond-N^+^ membrane (GE Healthcare, Uppsala, Sweden) by capillary transfer. Hybridization was performed at 58 °C overnight with biotin-labeled oligonucleotide probe. Washes and detection were carried out following the manufacturer’s instructions as described in Chemiluminescent Nucleic Acid Detection Module Kit (Thermo Fisher Scientific, Waltham, USA). Briefly, the membranes were blocked in blocking buffer for 15 min, and incubated with HRP-linked streptavidin (1:300) for 15 min with gentle shaking. The membranes were washed four times, then incubated with chemiluminescent substrate and exposed to X-ray film for 1–5 min. *GAPDH* was used as an internal control. The samples for blotting *GAPDH* were aliquoted before RNase R treatment and loaded separately in two wells for validation. The probe sequences were shown as below:

circNSUN2 junction probe (Supplementary 8a): 5′-AAGUUCUUCAGGAUACCUUAUGAUGAGGCCGCACGUUGAGGA-3′

*NSUN2*/circNSUN2 exon probe: 5′-GGCCGCACGUUGAGGAGCAGUGGUGGGAUCAUGCUAACAGCU-3′

*GAPDH* probe: 5′-UAUCCACUUUACCAGAGUUAAAAGCAGCCCUGGUGACCAGGCGCCCAAUACGACCAAA-3′

### Nuclear and cytoplasmic extraction

Cytoplasmic and nuclear fractions were isolated as described by the manufacturer, using the reagents supplied in PARIS™ Kit (AM1556, Thermo Fisher Scientific, Waltham, USA). Briefly, HCT116 was lysed in Cell Fraction Buffer on ice for 10 min. After centrifugation at 500 × *g* for 3 min at 4 °C, the supernatant was collected as cytoplasmic fraction. Followed by washing the pellet with Cell Fraction Buffer, the nuclei were collected.

### Total RNA extraction and RNA-SEQ

RNAs from control or sh-circNSUN2 HCT116 cells were used to generate mRNA Sequencing libraries using the NEB Next Ultra RNA Library Prep Kit (Illumina). RNA-SEQ libraries were then sequenced on the HiSeq 2000 platform according to the manufacturer’s recommendations by CapitalBio Technology (Beijing, China). The raw reads filtered by RNA-BisSeq data analysis method were mapped to the hg19 genome with HISAT2 (Johns Hopkins University, USA)^56^. The number of reads mapped to each Ensembl gene was counted using HiSeq sequencer^57^. The gene expression analyses were performed with Cuffquant and Cuffnorm (Cufflinks 2.2.1). Cuffdiff was used to analyze the DEGs between samples, fold change cutoff = 1.5.

### RNA fluorescence in situ hybridization (FISH)

Oligonucleotide-modified probe sequence for circNSUN2 and *HMGA2* was synthesized from Sangon Biotech (Shanghai, China). Fixed cells were washed in PBS, treated with RNase R at 37 °C for 15 min and then fixed again. The cell suspension was pipetted onto autoclaved glass slides, followed by dehydration with 70, 80 and 100% ethanol. Then hybridization was performed at 37 °C overnight in a dark moist chamber. After being washed twice in 50% formamide/2 × SSC for 5 min, the slices were incubated with the regents in Alexa Fluor^TM^ 488 Tyramide SuperBoost™ Kits (Thermo Fisher Scientific, Waltham, USA) for 30 min and sealed with parafilm containing DAPI. The images were acquired using a fluorescence microscopy (OLYMPUS FV1000 confocal microscopy, Japan). Fluorescence intensity was analyzed by ImageJ. Pearson’s correlation coefficient was analyzed by OLYMPUS FV1000 software. The probe sequences were shown as below:

circNSUN2: 5′-biotin labeling- AAGUUCUUCAGGAUACCUUAUGAUGAGGCCGCACGUUGAGGA-3′

*HMGA2*: 5′- Cy3- GGCAGACUCUUGUGAGGAUGUCUCUUCAGUUUCCUCCUGAGCAGGCUUC-3′

### Animal models

All mouse procedures were approved by the Institutional Animal Care and Use of SYSUCC. All of the BALB/c nude mice in each experimental group were obtained from Vital River Laboratory (Beijing, China). For the LM model, 2 × 10^6^ cells suspended in 40 μl PBS were injected into the inferior hemispleen into each 6-week-old BALB/c nude mouse. Eight weeks after cell injection, mice were euthanatized. For the lung metastasis model, 5 × 10^5^ cells suspended in 100 μl PBS were injected intravenously in the tail vein into each 4-week-old BALB/c nude mouse. Six weeks after cell injection, the mice were sacrificed.

The lungs and livers were removed and fixed with phosphate-buffered formalin. Subsequently, the numbers of metastatic nodules in the lungs and livers were carefully examined. For imaging tumors in live animals, VivoGloluciferin (Promega, Madison, WI) was dissolved in sterilized PBS (final concentration: 15 mg/ml). Mice were anaesthetized with isoflurane and injected intraperitoneally with 100 µl of the luciferin solution. After 15 min, images were acquired with the Xenogen IVIS Lumina series II for 5 min and analyzed using the LivingImage 2.11 software package (Xenogen Corp.).

### Transwell assays

For invasion assays, 2 × 10^5^ cells were planted in 500 μl of serum-free medium to a Boyden chamber (BD Biosciences, NewJersey, USA) in the insert of a 24-well plate. 1640 medium containing 30% FBS was added to the bottom chamber. After incubation for 24 h, the invaded cells in lower filters were fixed in methanol, stained in crystal violet (Sigma, MO, USA) and counted under microscope. All experiments were performed three times.

### Inverted invasion assays

Matrigel (BD biosciences, New Jersey, USA) was polymerized in transwell inserts for 1 h at 37 °C. The inserts were inverted and 4 × 10^5^ cells seeded directly onto the outer filter surface and allowed to adhere for 5 h. The inserts were placed in serum-free medium and the chamber was filled with 500 μl medium containing 10% FCS and 150 ng/ml IGF (Sino Biological, Beijing, China). At 36 h after seeding, cells were stained in medium containing 4 nM Calcein (Invitrogen, Carlsbad, USA) for 30 min at 37 °C and then visualized by confocal microscopy (OLYMPUS FV1000, Japan) using a ×40 objective lens. Optical sections were scanned at 10 μm intervals. The fluorescence intensity of each slice was quantified using ImageJ. The quantification of invading cells was determined by calculating the ratio of the sum of intensities of all slices beyond 30 µm compared to the total sum of intensity of all slides.

### 3D multicellular tumor spheroids invasion assay

The 3D coculture has been performed in 24-well dishes coated with Growth Factor-Reduced Matrigel (BD Biosciences, New Jersey, USA). Briefly, 1 × 10^4^ cells were embedded and were covered with growth medium supplemented with 2% Matrigel. The medium was exchanged every second days for 1 week. Bright-field images were acquired and the quantification of the invading area was analyzed using Olympus cellSens Standard 1.9.

### Immunofluorescence

CRC cells grown on the confocal dish (Corning, United States) were fixed with 4% paraformaldehyde in PBS for 20 min on ice and then permeabilized with 0.1% TritonX-100 in PBS for 10 min. After washing twice with PBS, cells were blocked with 5% BSA for 30 min at 37 °C and incubated with primary antibody overnight at 4 °C. The next day, cells were washed with PBS and then incubated with corresponding secondary antibody for 30 min at 37 °C, followed by staining with DAPI for nucleus staining. Fluorescent images were acquired using OLYMPUS FV1000 confocal microscopy. The relative fluorescence densities were analyzed by ImageJ, and plotted using GraphPad Prism 6 software.

### RNA pull down

The biotin-coupled RNA complex was pulled down by incubating the cell lysates with streptavidin-coated magnetic beads (Invitrogen, Carlsbad, USA) following the manufacturer’s instructions. The enrichment of circNSUN2 or *HMGA2* in the capture fractions was evaluated by qRT-PCR analysis. The bound proteins were eluted from the packed beads and analyzed by SDS-PAGE.

circNSUN2 junction probe (Supplementary 8a):

5′-AAGUUCUUCAGGAUACCUUAUGAUGAGGCCGCACGUUGAGGA-3′

Control probe (ordered from Sangon Biotech, Shanghai, China):

5′-UUGUACUACACAAAAGUACUG-3′

The proteins in the capture complex were identified by western blotting or silver staining or mass spectrometry analysis.

### Silver staining and mass spectrometry analysis

Silver staining was performed using the Fast Silver Stain Kit (Beyotime, Haimen, China) as the protocol described, while MS was done by Wininnovate Bio (Shenzhen, China). Afterwards, protein identification and quantification were accomplished by Proteome Discoverer software (version 1.4; Thermo Fisher Scientific, USA). A protein with area ratio (circNSUN2/CTRL) > 2, unique peptides > 2 was considered significant.

### RNA-binding protein immunoprecipitation (RIP)

RIP assay was performed by using a Magna RIP RNA-Binding Protein Immunoprecipitation Kit (Millipore), according to the manufacturer’s instructions. Briefly, HCT116 cells were harvested after 48 h post transfection and lysed in RIP lysis buffer on ice for 30 min. After centrifugation, the supernatant was incubated with 30 μl of Protein-A/G agarose beads (Roche, USA) and antibodies. After overnight incubation, the immune complexes were centrifuged then washed six times with washing buffer. The beads-bound proteins were further analyzed using western blotting. The immunoprecipitated RNA was applied to qRT-PCR analysis.

### Protein purification

293T cells were transfected with Flag-tagged plasmid constructs and after 36 h harvested with lysis buffer followed by sonication on ice. After centrifugation at 13,800 × *g* for 20 min, the supernatant was filtered using 0.2 μm syringe filter and then incubated with anti-Flag M2 (Sigma, California, USA) by gently rotating overnight at 4 °C. The beads were then washed twice with Lysis buffer and twice with TBS and incubated with 3 × Flag-Peptide (APExBIO, Huston, USA) by gently rotating for 1 h at 4 °C to elute the bound proteins. The elution was done twice to maximize the recovery. The protein thus purified was condensed.

### Electrophoretic mobility shift assay (EMSA)

Biotin-labeled RNA oligonucleotides were obtained from Sangon Biotech (Shanghai, China). RNA EMSA assay was carried out using LightShift™ Chemiluminescent RNA EMSA Kit (Thermo Fisher Scientific, Waltham, USA), according to the manufacturer’s instructions. Briefly, purified proteins were incubated with biotin-labeled oligonucleotide probe in binding buffer for 30 min at room temperature. The RNA–protein complexes were resolved by electrophoresis on 6% nondenaturing polyacrylamide gel in 0.5× TBE buffer at 100 V for 1 h, then transferred to a nylon membrane at 400 mA (~100 V) for 30 min. The membrane was crosslinked, blocked in blocking buffer for 15 min, and incubated with HRP-linked streptavidin (1:300) for 15 min with gentle shaking. The membranes were incubated with chemiluminescent substrate and exposed to X-ray film for 1–5 min. For supershift assays, recombinant proteins were preincubated with antibodies at 0 °C for 20 min followed by the addition of the labeled probe. The probe sequences were shown as below:

For YTHDC1,

circNSUN2—m^6^A Wild type: 5′-AUAAGGUAUCCUGAAGAACU-3′-Biotin

circNSUN2—m^6^A Mutant type: 5′-AUAAGGUAUCCUGAAGAGCU-3′-Biotin

For IGF2BP2,

circNSUN2—Wild type: 5′-CCUCAUCAUAAGGUAUCCUG-3′-Biotin

circNSUN2—Mutant type: 5′-CCUCACCACAAGGUAUCCUG-3′-Biotin

### Luciferase reporter assay

The sequence of *HMGA2* was cloned downstream of p-GLO Dual-Luciferase vector (Vigenebio, Maryland, USA). Mutations were performed in the binding sites. HCT116 cells were seeded into 24-well dishes at a density of 30% confluency per well 24 h before transfection and cotransfected with a mixture of 800 ng p-GLO Dual-Luciferase reporter and 20 pmol siRNA. After 48 h, the relative luciferase activity was measured as the ratio between Firefly and Renilla luciferase activities with a dual luciferase reporter assay system (Promega, Madison, WI). Rellina luciferase activity was used as an internal control. For circNSUN2 knockdown or overexpression group, the relative Luc/Rluc ratio was further normalized to that of control sample.

### Statistical analysis

All experiments were carried out at least three times and data from one representative experiment are shown. Data are presented as mean ± standard deviation (S.D.). The statistical significance of differences was evaluated by two-tailed Student’s *t* test or two-way ANOVA. OS were assessed with the Kaplan−Meier method and compared by the Log-rank test. The correlations among circNSUN2, *HMGA2* and *CXCR4* expression in CRC patients were calculated by Pearson correlation analysis and *P* value computed using the R language function. *P* values < 0.05 were considered statistically significant. All statistical analyses were carried out using R or GraphPad Prism (version 6.0).

### Reporting summary

Further information on research design is available in the Nature Research Reporting Summary linked to this article.

## Supplementary information

Supplementary Information

Reporting Summary

Description of Additional Supplementary Files

Supplementary Dataset 1

Supplementary Dataset 2

Supplementary Dataset 3

Supplementary Dataset 4

Source Data

## Data Availability

A reporting summary for this Article is available as a Supplementary Information file. Microarray data are accessible at the Gene Expression Omnibus (GEO) under accession number: GSE121895. RNA-SEQ data have been deposited in the Sequence Read Archive (SRA) database under project PRJNA549980, ID: SRP202052. The mass spectrometry data are accessible using PeptideAtlas Dataset ID: PASS01424. The source data underlying Figs. 2b, d−e, 3a–d, 4b, c, f, g, 5b, d, 6a−c, e, f, and 7a−e and Supplementary Figs. 1a−d, 2a−h, 3a−f, 4a−h, 5b−f, 6a−c and 7a−d are provided as a Source Data file. Unprocessed gel images for Figs. 2c, 4a, b, d, 5a, b, d, e, Supplementary Figs. 4a, b, 5b, c, f have been provided in Supplementary Fig. 9. Other data that support the findings of this study are available from the corresponding authors on reasonable request.
