# Supplementary material for: N6-methyladenosine modification of circNSUN2 facilitates cytoplasmic export and stabilizes HMGA2 to promote colorectal liver metastasis
Source: Nat Commun. 2019 Oct 16;10:4695. doi: 10.1038/s41467-019-12651-2 (PMC6795808; doi:10.1038/s41467-019-12651-2)
Supplement: Supplementary file 1 — Supplementary Information [file 41467_2019_12651_MOESM1_ESM.pdf]

Supplementary Information

*N*6-methyladenosine modification of circNSUN2 facilitates cytoplasmic export and stabilizes *HMGA2* to promote colorectal liver metastasis

Chen et al.

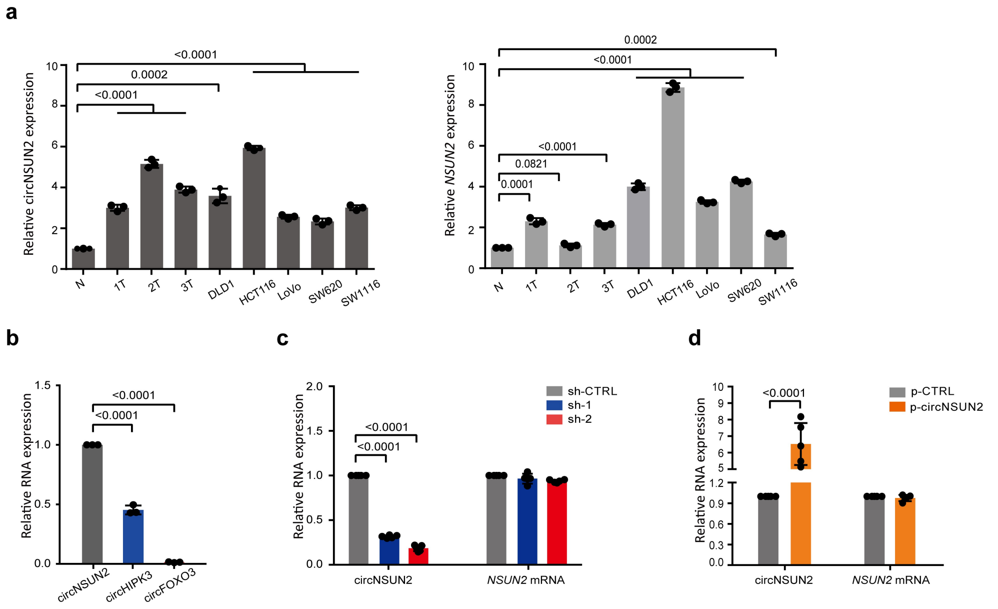

Supplementary Figure 1. Constructions of stable circNSUN2-knockdown and circNUSN2-overexpression CRC cell line. (a) qRT-PCR for the abundance of circNSUN2 (left panel) and *NSUN2* mRNA (right panel) in three CRC tissues and five CRC cell lines. Data represent mean ± S.D. from three independent experiments; dot plot reflects data points from independent experiment. The *P* values were determined by a two-tailed unpaired Student's *t*-test. (b) qRT-PCR for circNSUN2, circHIPK3 and circFOXO3 in CRC cells. Data represent mean ± S.D. from three independent experiments; dot plot reflects data points from independent experiment. The *P* values were determined by a two-tailed unpaired Student's *t*-test. (c) qRT-PCR for circNSUN2 and *NSUN2* mRNA in CRC cells treated with two shRNAs as described. (d) qRT-PCR for circNSUN2 and *NSUN2* mRNA in CRC cells transfected with control vector or circNSUN2 overexpression plasmid. For (c) and (d), Data represent mean ±S.D. from five independent experiments; dot plot reflects data points from independent experiment. The *P* values were determined by a two-tailed unpaired

Student's *t*-test. Source data are provided as a Source Data file.

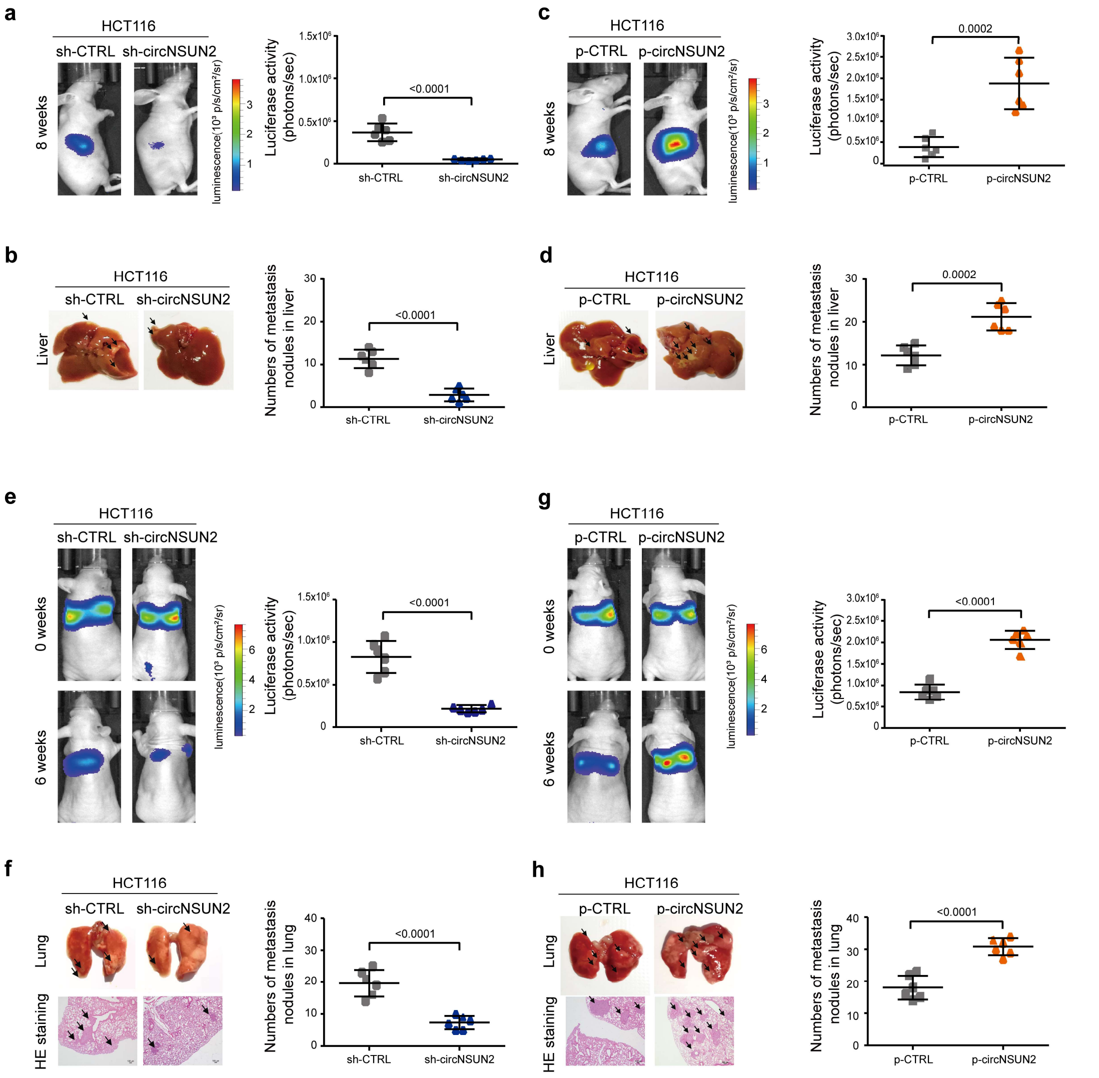

Supplementary Figure 2. CircNSUN2 promotes metastasis of CRC. (a-d) Decreased (a, b) or increased (c, d) tumor metastasis formed in the livers of mice through the inferior hemispleen implantation of circNSUN2-konckdown (a, b) or circNSUN2-overexpression (c, d) HCT116 cells. (a, c) Left, representative bioluminescent images of livers for each experimental group at 8 weeks. Right, statistical analysis of bioluminescent tracking plots. (b, d) Left, representative liver. Right, the number of metastatic nodules formed in the lungs of mice for each group (n = 6 mice / group). Data represent mean ± S.D.; dot plot reflects data points from

independent experiment. The *P* values were determined by a two-tailed unpaired Student's *t*-test. (e-h) Decreased (e, f) or increased (g, h) tumor metastasis formed in the lungs of mice through vein tail injection of circNSUN2-konckdown (e, f) or circNSUN2-overexpression (g, h) HCT116 cells. (e, f) Left, representative bioluminescent images of lungs for each experimental group during 6 weeks. Right, statistical analysis of bioluminescent tracking plots. (f, h) Left, representative lung and representative HE staining of lung metastatic lesions, Original magnification, 4×, scale bar, 100 μm. Right, the number of metastatic nodules formed in the lungs of mice for each group (n = 6 mice / group). Data represent mean ± S.D.; dot plot reflects data points from independent experiment. The *P* values were determined by a two-tailed unpaired Student's *t*-test. Source data are provided as a Source Data file.

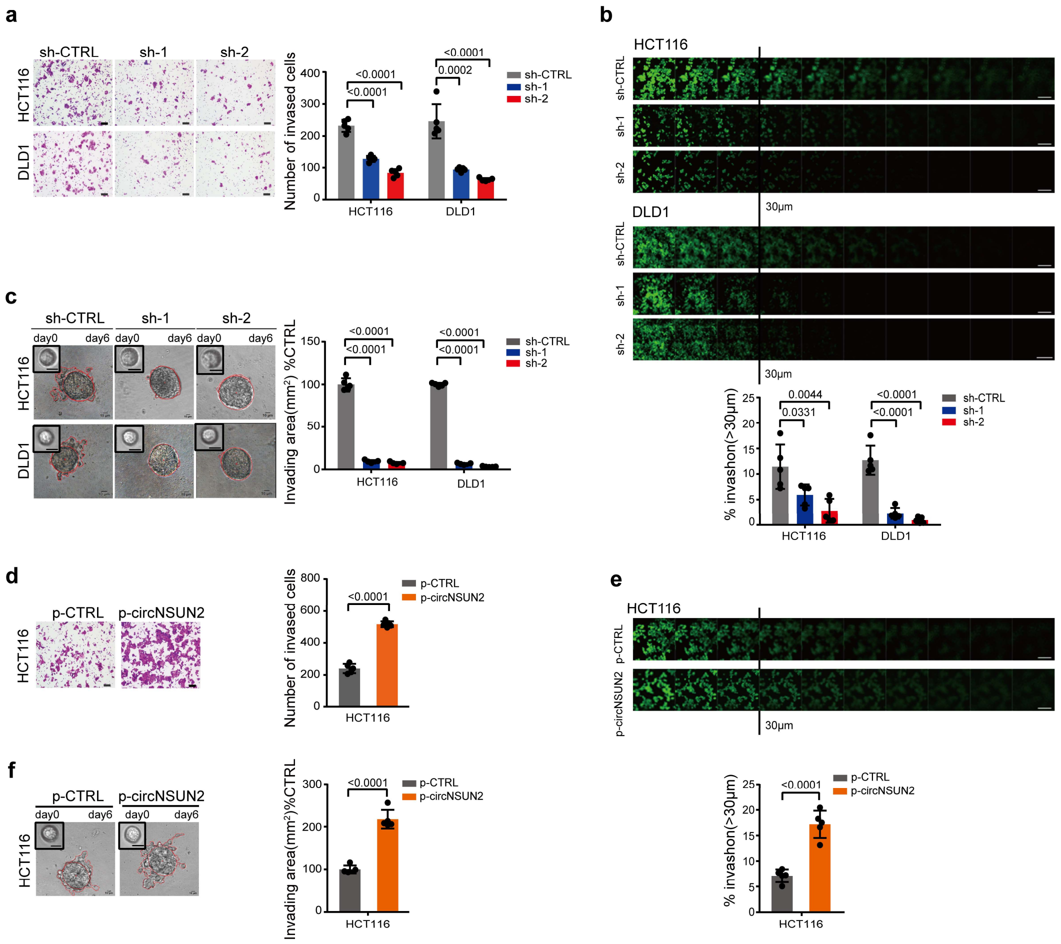

Supplementary Figure 3. CircNSUN2 promotes metastasis of CRC *in vitro*. (a) Transwell assay showing that knockdown of circNSUN2 inhibited the invasion ability of indicated CRC cells. Left, representative images. Scale bar, 100 μm. Right histograms of invasive cell numbers. (b) Inverted invasion assay showing that depletion of circNSUN2 inhibited the invasion ability of the indicated CRC cells. Top, representative images. Scale bar, 100 μm. Bottom, histograms of invasive cell ratios. (c) 3D multicellular tumor spheroids invasion assay showing that knockdown of circNSUN2 inhibited the invasion capacity of indicated CRC cells. Red dotted line indicates the spheroid body and invading area. Left, representative images of day0 and day6. Scale bar, 10μm. Right, histograms represent the quantification of the

invading area and protrusion. (d) Transwell assay showing that overexpression of circNSUN2 accelerated the invasion of the indicated CRC cells. Left, representative images. Scale bar, 100 μm. Right, histograms of invasive cell numbers. (e) Inverted invasion assay showing that overexpression of circNSUN2 enhanced the invasion capacity of the indicated CRC cells. Top, representative images. Scale bar, 100 μm. Bottom, histograms of invasive cell ratios. (f) 3D multicellular tumor spheroids invasion assay showing that overexpression of circNSUN2 promoted the invasion of the indicated CRC cells. Red dotted line indicates the spheroid body and invading area. Left, representative images of day0 and day6. Scale bar, 10μm. Right, histograms represent the quantification of the invading area and protrusion.

For (a-f), data represent mean ± S.D. from five independent experiments; dot plot reflects data points from independent experiment. The *P* values were determined by a two-tailed unpaired Student's *t*-test. Source data are provided as a Source Data file.

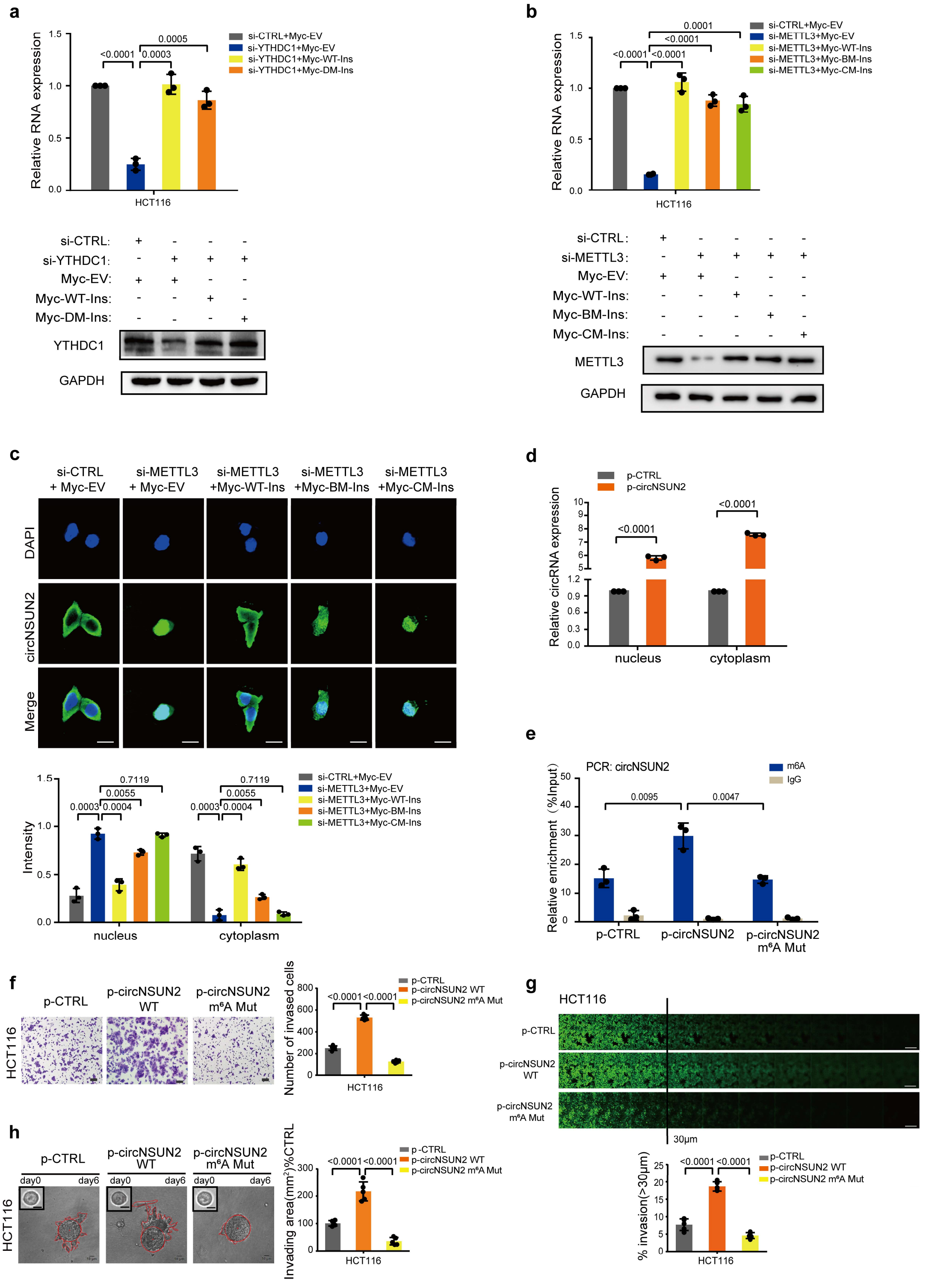

Supplementary Figure 4. *N6*-methyladenosine modification of circNSUN2 facilitates cytoplasmic export. (a) qRT-PCR analysis (top panel) for *YTHDC1* mRNA expression and western blotting (bottom panel) for YTHDC1 protein

expression in HCT116 indicated cells. Data represent mean ± S.D. from three independent experiments; dot plot reflects data points from independent experiment. The *P* values were determined by a two-tailed unpaired Student's *t*-test. (b) qRT-PCR analysis (top panel) for *METTL3* mRNA expression and western blotting (bttom panel) for METTL3 protein expression in HCT116 cells. Data represent mean ± S.D. from three independent experiments; dot plot reflects data points from independent experiment. The *P* values were determined by a two-tailed unpaired Student's *t*-test. (c) RNA-FISH showing that the increased nuclear staining of circNSUN2 caused by METTL3 RNAi was rescued by overexpression of WT but not the mutant METTL3. Scale bar, 10 μm. Data represent mean ± S.D. from three independent experiments; dot plot reflects data points from independent experiment. The *P* values were determined by a two-tailed unpaired Student's *t*-test. (d) Cytoplasmic and Nuclear mRNA Fractionation experiment showing that cytoplasmic circNSUN2 contents were greatly increased after its overexpression. *β-actin* and *U3* were applied as positive controls in the cytoplasm and nucleus, respectively. Data represent mean ± S.D. from three independent experiments; dot plot reflects data points from independent experiment. The *P* values were determined by a two-tailed unpaired Student's *t*-test. (e) MeRIP assay showing that m^6A methylation levels was increased upon circNSUN2 overexpression. Data represent mean ± S.D. from three independent experiments; dot plot reflects data points from independent experiment. The *P* values were determined by a two-tailed unpaired Student's *t*-test. Transwell assay (f), Inverted invasion assay (g) and 3D multicellular tumor spheroids invasion assay (h)

showing that overexpression of circNSUN2 m^6A-binding mutant attenuated HCT116 cells invasion activity. Scale bar, 100 µm (f), 100 µm (g) and 10 µm (h). Data represent mean ± S.D. from five independent experiments; dot plot reflects data points from independent experiment. The *P* values were determined by a two-tailed unpaired Student's *t*-test. Source data are provided as a Source Data file. Unprocessed original scans of blots are shown in Supplementary Figure 10.

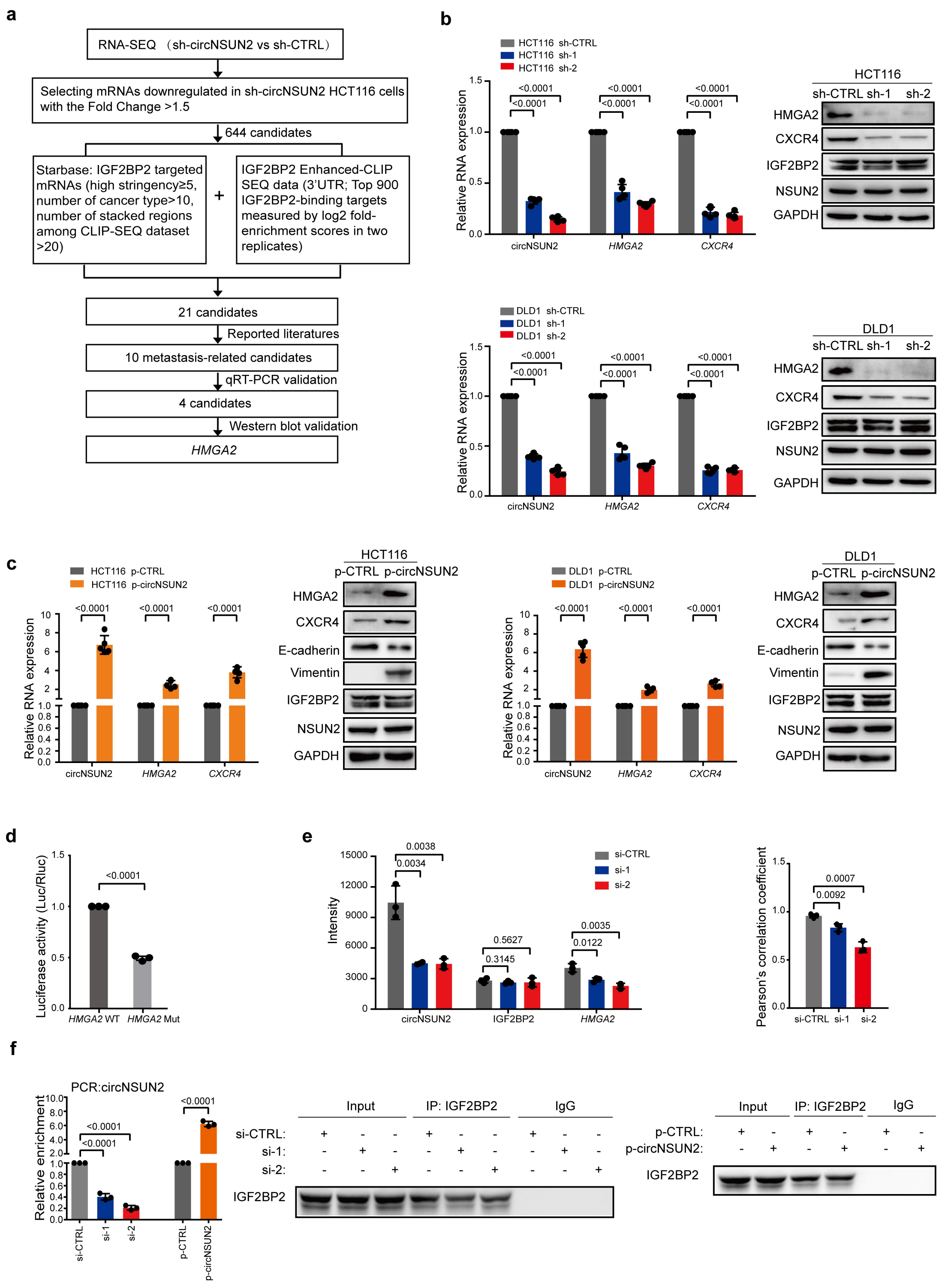

Supplementary Figure 5. CircNSUN2 binds to IGF2BP2 and enhances its interaction with *HMGA2*. (a) Flowing chart illustrates the criteria of identifying of *HMGA2* as the target of circNSUN2. (b-c) qRT–PCR analysis (left panel) for the

RNA expression of circNSUN2, *HMGA2* and *CXCR4*. Western blotting analysis (right panel) for the protein expression of HMGA2, CXCR4, E-cadherin, Vimentin, IGF2BP2, NSUN2 in control and circNSUN2-knockdown (b) or circNSUN2-overexpression (c) CRC cells. GAPDH was used as an internal control. Data represent mean ± S.D. from five independent experiments; dot plot reflects data points from independent experiment. The *P* values were determined by a two-tailed unpaired Student's *t*-test. (d) Relative luciferase activity of luciferase reporter gene with *HMGA2*-WT or *HMGA2*-Mut in HCT116 cells. Data represent mean ± S.D. from three independent experiments; dot plot reflects data points from independent experiment. The *P* value was determined by a two-tailed unpaired Student's *t*-test. (e) Fluorescence intensity analysis of the expression of circNSUN2, IGF2BP2 and *HMGA2* (left lanel). Pearson's correlation coefficient analysis of IF-FISH showing that knockdown of circNSUN2 attenuated the colocalization of *HMGA2* and IGF2BP2 (right panel). Data represent mean ± S.D. from three independent experiments; dot plot reflects data points from independent experiment. The *P* values were determined by a two-tailed unpaired Student's *t*-test. (f) qRT–PCR analysis for the RNA expression of circNSUN2 from RIP assays upon circNSUN2 knockdown or overexpression. Data represent mean ± S.D. from three independent experiments; dot plot reflects data points from independent experiment. The *P* values were determined by a two-tailed unpaired Student's *t*-test. Western blotting showing IGF2BP2 expression from RIP assays upon circNSUN2 knockdown or overexpression. Source

data are provided as a Source Data file. Unprocessed original scans of blots are shown in Supplementary Figure 10.

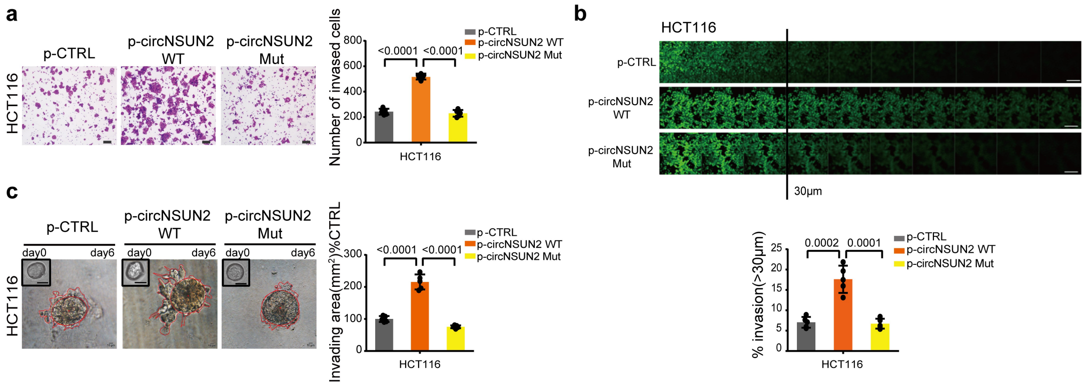

Supplementary Figure 6. CircNSUN2 promotes LM metastasis of CRC through HMGA2 pathway. Transwell assay (a), Inverted invasion assay (b) and 3D multicellular tumor spheroids invasion assay (c) showing that overexpression of circNSUN2 mutant caused reduced migration and invasive abilities in HCT116 cells. Scale bar, 100 µm (a), 100 µm (b) and 10 µm (c). Data represent mean ± S.D. from five independent experiments; dot plot reflects data points from independent experiment. The *P* values were determined by a two-tailed unpaired Student's *t*-test. Source data are provided as a Source Data file.

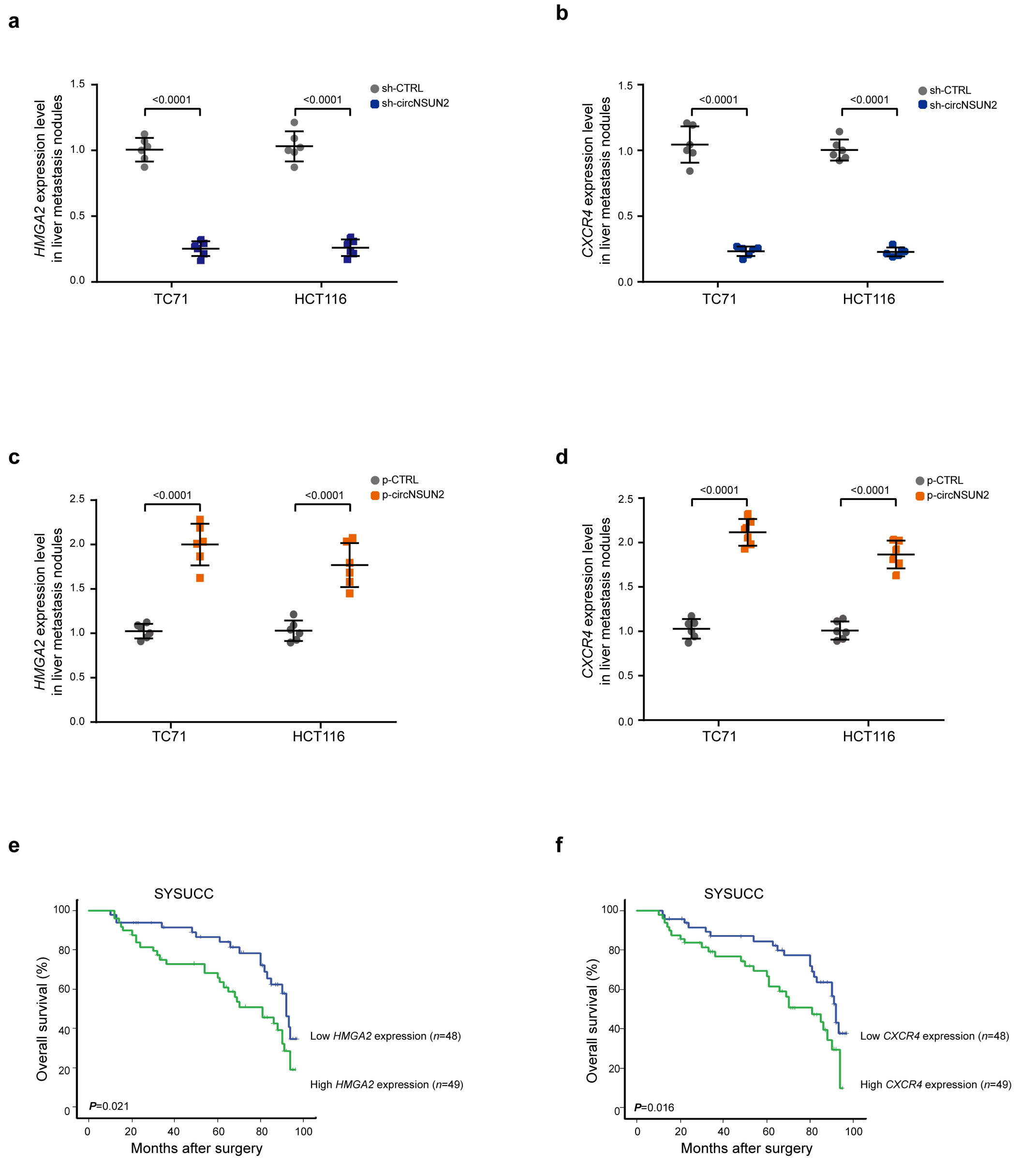

Supplementary Figure 7. CircNSUN2 promotes LM metastasis of CRC through HMGA2 pathway. (a, b) Decreased expression of *HMGA2* (a) and *CXCR4* (b) in the liver metastasis nodules of mice through hemispleen injection of circNSUN2-knockdown CRC cells. (c, d) Increased expression of *HMGA2* (c) and *CXCR4* (d) in the liver metastasis nodules of mice through hemispleen injection of circNSUN2-overexpression CRC cells (n = 6 mice / group). Data represent mean ± S.D.; dot plot reflects data points from independent experiment. The *P* values were

determined by a two-tailed unpaired Student's *t*-test. (e, f) Kaplan-Meier analyses of OS in CRC patients with low versus high expression the *HMGA2* and *CXCR4* from SYSUCC cohorts. The *P* values were determined by a Log-rank test. Source data are provided as a Source Data file.

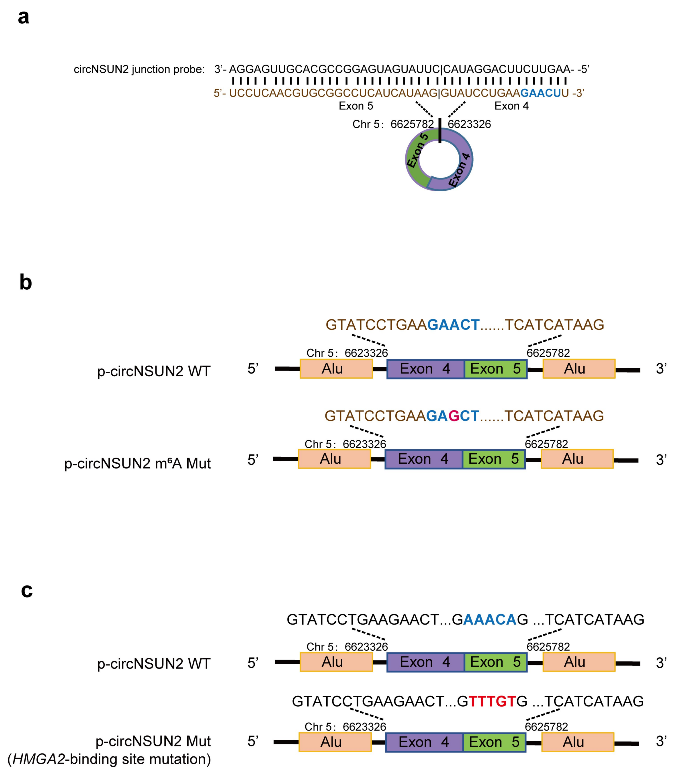

Supplementary Figure 8. Schematic illustration of the circNSUN2 wild-type or m^{6}A (a) or *HMGA2* binding site mutant (b) constructs. The red letters represent the mutant sites.

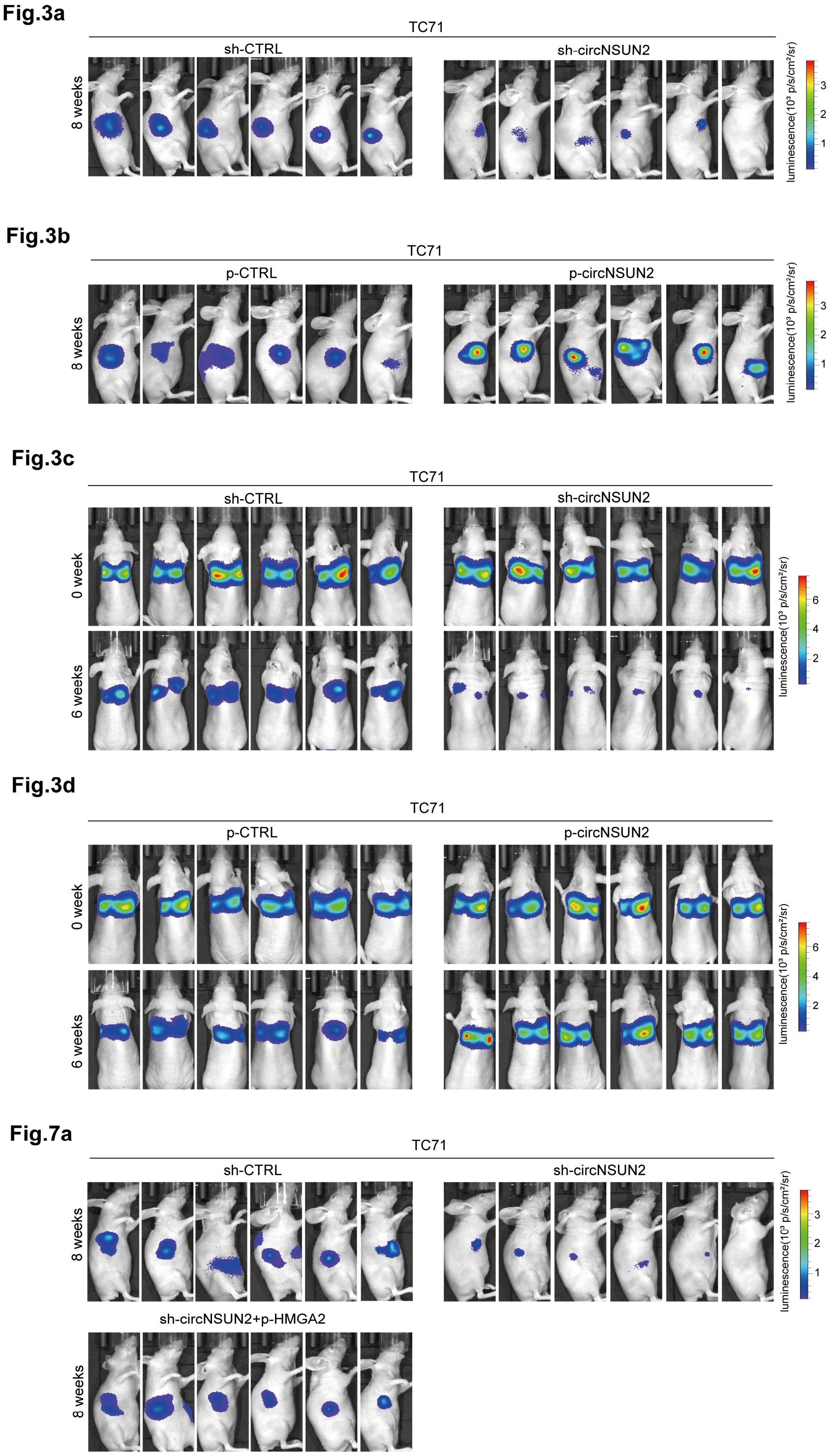

Supplementary Figure 9. The images for all animals of Fig.3 and Fig. 7.

Fig. 2c

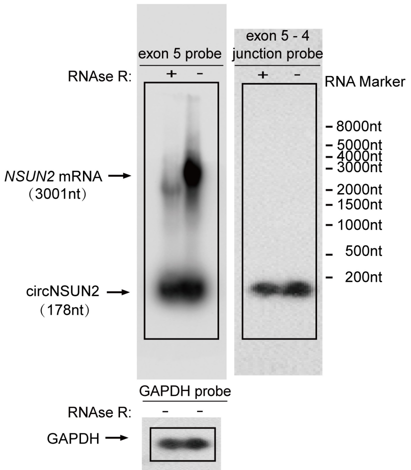

Fig. 4a

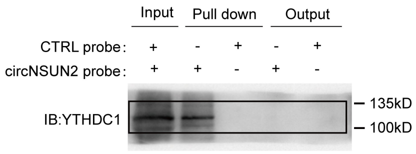

Fig. 4b

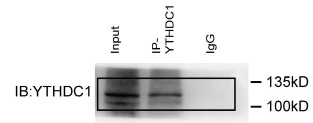

Fig. 4d

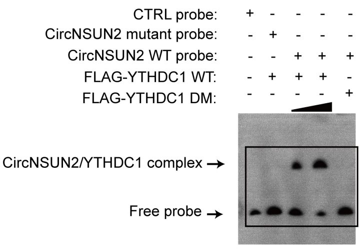

Fig. 5a

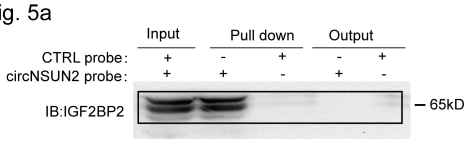

Fig. 5b

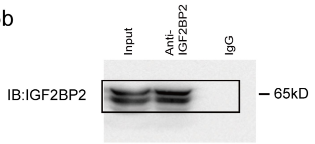

Fig. 5d

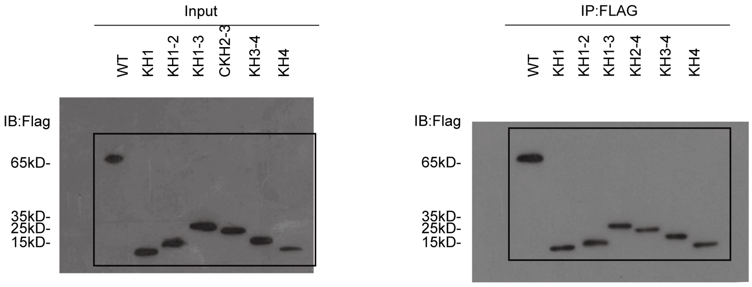

Fig. 5e

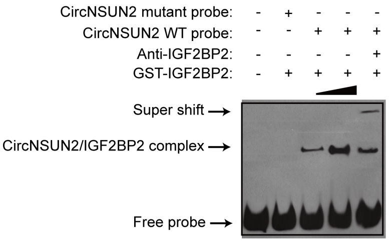

Supplementary Fig. 4a

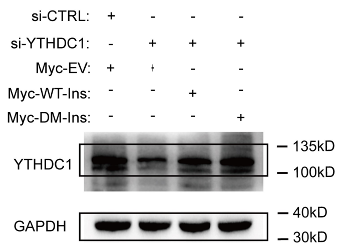

Supplementary Fig. 4b

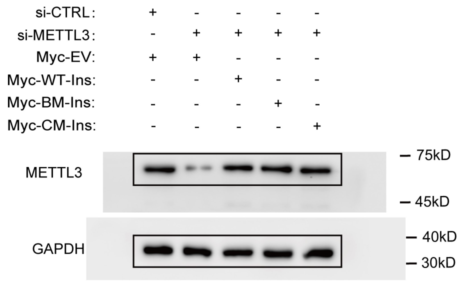

Supplementary Fig. 5b

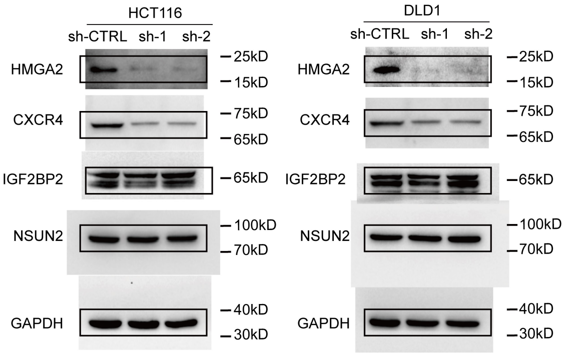

Supplementary Fig. 5c

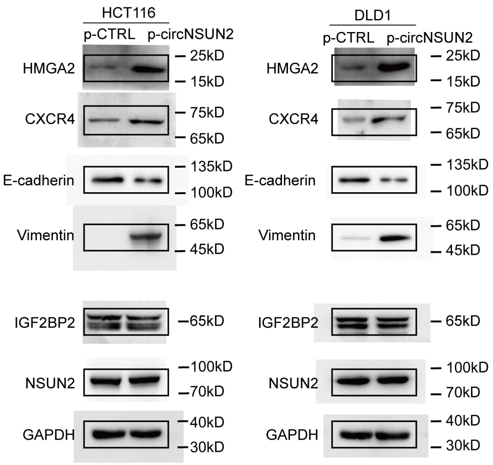

Supplementary Fig. 5f

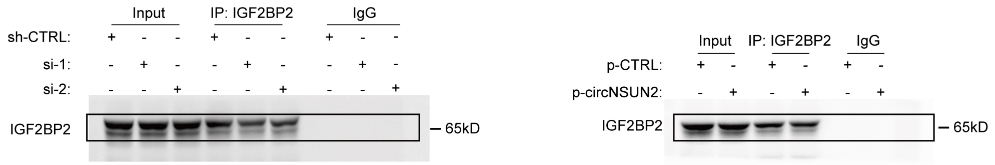

Supplementary Figure 10. Full blots of figures.

The black sections indicate blot results shown in the indicated figures.

Supplementary table 1. Relationship between circNSUN2 expression level and clinicalpathological parameters of CRC

Variable	Number of cases	circNSUN2 expression		*P**
		Low expression	High expression	
**Age, yr**		48	49	
< 60	46	24	22	
≥ 60	51	24	27	0.615
**Gender**				
Male	49	25	24	
Female	48	23	25	0.760
**Tumor location**				
Rectum	47	20	27	
Colon	50	28	22	0.186
**Histological grade(WHO)**				
G1-2	66	36	30	
G3	31	12	19	0.146
**pT status**				
T1-T2	29	18	11	
T3-T4	68	30	38	0.105
**Clinical stage**				
III	52	19	33	
I+II	45	29	16	0.006
**Lymph node status**				
Metastasis	52	19	33	
No metastasis	45	29	16	0.006
**CEA level**				
< 5(ng/ml)	55	29	26	
≥ 5(ng/ml)	42	19	23	0.465

CRC, colorectal carcinoma; CEA, carcino-embryonicantigen; * χ^2 text

Supplementary Table 2. Univariate and multivariate Cox regression analysis of different prognostic variables in patients of CRC

Variable	Subset	Hazard ratio for DSS (95% CI)	*P* value
Univariate analysis (*n*=97)			
Age (yr)	< 60 *vs*.≥ 60	1.349 (0.763-2.386)	0.340
Gender	Male *vs*. Female	1.478 (0.831-2.631)	0.184
Tumor location	Colon *vs*. Rectum	1.204 (0.685-2.118)	0.519
Histological grade (WHO)	G1-2 *vs*. G3	1.831(1.034-3.243)	0.038
Clinical stage	I+II *vs*. III	2.210 (1.238-3.944)	0.007
pT status	T1+2 *vs*. T3+4	2.934(1.506-5.713)	0.002
pN status	N0 *vs*. N1+2	2.210 (1.238-3.944)	0.007
CEA level	< 5(ng/ml) *vs*. ≥ 5(ng/ml)	1.479 (0.836-2.616)	0.179
circNSUN2 expression level	Low expression *vs*. High expression	2.776 (1.535-5.018)	0.001
Multivariate analysis (*n*=97)			
Histological grade (WHO)	G1-2 *vs*. G3	1.543 (0.858-2.774)	0.147
pT status	T1+2 *vs*. T3+4	2.040(1.016-4.094)	0.045
pN status	N0 *vs*. N1+2	1.416(0.760-2.639)	0.273
circNSUN2 expression level	Low expression *vs*. High expression	2.094 (1.123-3.904)	0.020

CRC, colorectal carcinoma; CEA, carcino-embryonicantigen;

Supplementary Table 3. Clinicopathological characteristics of patients with CRC with liver metastasis

Characteristics	Number of patients
Age, yr	
< 60	16
≥ 60	9
Gender	
Male	19
Female	6
Tumor location	
Colon	18
Rectum	7
Histological grade(WHO)	
G1-2	22
G3	3
CEA level	
< 5(ng/ml)	13
≥ 5(ng/ml)	12
Corresponding liver metastasis	
yes	20
no	5

CRC, colorectal carcinoma; CEA, carcino-embryonicantigen;

Supplementary Table 4. List of top 15 candidates of circNSUN2-interacting proteins that were identified by RNA pull down and MS

Gene Name	Unique Peptides	Coverage (%)	Molecular Weight (kDa)	Accession
IGF2BP2	21	42	66.1	Q9Y6M1
IGF2BP3	10	24	63.7	O00425
RPL6	8	32	32.7	Q02878
HNRNPC	8	23	33.7	P07910
DDX21	9	12	87.3	Q9NR30
MOV10	12	15	113.7	Q9HCE1
ILF2	9	29	43.1	Q12905
PABPC4	5	14	70.8	Q13310
DHX9	8	7	141.0	Q08211
RPL38	4	50	8.2	P63173
RPL18A	6	21	20.8	Q02543
MAGEB2	5	16	35.3	O15479
RPL36	3	21	12.2	Q9Y3U8
YTHDC1	6	9	84.7	Q96MU7
ATP5O	3	17	23.3	P48047

Supplementary Table 5. Candidates of potential genes regulated by circNSUN2 in CRCs

Characteristics	Number	Gene Name
Genes downregulated in circNSUN2 knockdown CRC cells with Fold Change >1.5 and bound by IGF2BP2	21 Genes	*ACVR1*, *ANP32A*, *CAP1*, *CERS6*, *FYTTD1*, *GLO1*, *HMGA2*, *JAK1*, *LDHA*, *MCFD2*, *MMGT1*, *MTMR2*, *NDC1*, *PEG10*, *PRKACB*, *RBPJ*, *TBC1D23*, *TCF12*, *TIMP3*, *TMEM106B*, *TSEN15*
Genes associated with metastasis	10 Genes	*ANP32A*, *CAP1*, *HMGA2*, *JAK1*, *MCFD2*, *MTMR2*, *RBPJ*, *PEG10*, *TCF12*, *TMEM106B*
