# Supplementary material for: N6-methyladenosine modification of circNSUN2 facilitates cytoplasmic export and stabilizes HMGA2 to promote colorectal liver metastasis
Source: Nat Commun. 2019 Oct 16;10:4695. doi: 10.1038/s41467-019-12651-2 (PMC6795808; doi:10.1038/s41467-019-12651-2)
Supplement: Supplementary file 2 — Reporting Summary [file 41467_2019_12651_MOESM2_ESM.pdf]

nature research

Corresponding author(s): Dan Xie

Reporting Summary

Nature Research wishes to improve the reproducibility of the work that we publish. This form provides structure for consistency and transparency in reporting. For further information on Nature Research policies, see Authors & Referees and the Editorial Policy Checklist.

Statistical parameters

When statistical analyses are reported, confirm that the following items are present in the relevant location (e.g. figure legend, table legend, main text, or Methods section).

n/a	Confirmed	
☐	☒	The exact sample size (n) for each experimental group/condition, given as a discrete number and unit of measurement
☐	☒	An indication of whether measurements were taken from distinct samples or whether the same sample was measured repeatedly
☐	☒	The statistical test(s) used AND whether they are one- or two-sided<br>*Only common tests should be described solely by name; describe more complex techniques in the Methods section.*
☒	☐	A description of all covariates tested
☐	☒	A description of any assumptions or corrections, such as tests of normality and adjustment for multiple comparisons
☐	☒	A full description of the statistics including central tendency (e.g. means) or other basic estimates (e.g. regression coefficient) AND variation (e.g. standard deviation) or associated estimates of uncertainty (e.g. confidence intervals)
☐	☒	For null hypothesis testing, the test statistic (e.g. F, t, r) with confidence intervals, effect sizes, degrees of freedom and P value noted<br>*Give P values as exact values whenever suitable.*
☒	☐	For Bayesian analysis, information on the choice of priors and Markov chain Monte Carlo settings
☒	☐	For hierarchical and complex designs, identification of the appropriate level for tests and full reporting of outcomes
☐	☒	Estimates of effect sizes (e.g. Cohen's d, Pearson's r), indicating how they were calculated
☐	☒	Clearly defined error bars<br>*State explicitly what error bars represent (e.g. SD, SE, CI)*

Our web collection on statistics for biologists may be useful.

Software and code

Policy information about availability of computer code

Data collection	For CircRNA Microarray analysis,scanned images were imported into Agilent Feature Extraction software (G2505C) for raw data extraction. For RNA-SEQ analysis, mRNA Sequencing libraries were generated using the NEB Next Ultra RNA Library Prep Kit (Illumina) and then sequenced on the HiSeq 2000 platform. For Mass spectrometry analysis, protein identification were accomplished by Proteome Discoverer software (version 1.4). For imaging tumors in live animals, images were acquired with the Xenogen IVIS Lumina series II. For Immunofluorescence and RNA fluorescence in situ hybridization, fluorescent images were acquired using OLYMPUS FV1000 confocal microscopy. Luciferase activity was measured with a dual luciferase reporter assay system (Promega).

Data analysis	For CircRNA Microarray analysis， quantile normalization of raw data was performed using the R software limma package （version 3.38.2). Subsequent circRNAs differential expression profiling was performed by the R software edgeR package (version 3.24.0). The Average Normalized Fold Changes were calculated by the R software limma package. For RNA-SEQ analysis, the gene expression analyses were performed with Cuffquant and Cuffnorm (Cufflinks 2.2.1). Cuffdiff was used to analyze the DEGs between samples. For imaging tumors in live animals, images were analyzed using the LivingImage 2.11 software package (Xenogen Corp.) For Mass spectrometry analysis, protein identification and quantification were accomplished by Proteome Discoverer software (version 1.4). For Immunofluorescence and RNA fluorescence in situ hybridization, fluorescence intensity was analyzed by Image J. Quantification of Matrigel images was performed in ImageJ, in which the intensity of each slice was measured. Protein identification and quantification were accomplished by Proteome Discoverer software (version 1.4).Statistical analysis in SYSUCC cohorts was carried out with GraphPad Prism (version 6.0). The correlations among circNSUN2, HMGA2 and CXCR4 expression in CRC patients were calculated by Pearson correlation analysis and p value computed using the R language function (version 3.5.1). All statistical analyses were carried out using R or GraphPad Prism.

For manuscripts utilizing custom algorithms or software that are central to the research but not yet described in published literature, software must be made available to editors/reviewers upon request. We strongly encourage code deposition in a community repository (e.g. GitHub). See the Nature Research guidelines for submitting code & software for further information.

Data

Policy information about availability of data

All manuscripts must include a data availability statement. This statement should provide the following information, where applicable:

- Accession codes, unique identifiers, or web links for publicly available datasets
- A list of figures that have associated raw data
- A description of any restrictions on data availability

Microarray data are accessible at the Gene Expression Omnibus under accession number: GSE121895. RNA-SEQ data have been deposited in the Sequence Read Archive database under project PRJNA549980, ID: SRP202052. The mass spectrometry data are accessible using PeptideAtlas Dataset ID: PASS01424.

Field-specific reporting

Please select the best fit for your research. If you are not sure, read the appropriate sections before making your selection.

☒ Life sciences ☐ Behavioural & social sciences ☐ Ecological, evolutionary & environmental sciences

For a reference copy of the document with all sections, see nature.com/authors/policies/ReportingSummary-flat.pdf

Life sciences study design

All studies must disclose on these points even when the disclosure is negative.

Sample size	In general, no statistical methods were used to predetermine sample size. For the animal experiments, sample size was determined based on previous experience with models of colorectal cancer and literature reports. For human samples, samples were collected until we felt the sample size was sufficient to give reliable estimates. For cell and biochemical data, we aimed to collect data from three biological replicates when possible.
Data exclusions	No data were excluded from the analyses
Replication	Results were confirmed in at least three biological replicates for each experiment unless otherwise stated.
Randomization	Samples/mice/human subjects were not randomized. Controlling for covariates was unnecessary for the human samples as all comparisons were between paired tumor/normal samples collected from the same patient. We did not control for covariates in the animal experiments because all animals were the same age and sex and were purchased from the same supplier.
Blinding	For the animal experiments, investigators were blinded to group allocation during intraperitoneal injections and when assessing outcome. For the other experiments on human samples and cell lines, blinding was not performed due to feasibility.

Behavioural & social sciences study design

All studies must disclose on these points even when the disclosure is negative.

Study description	*Briefly describe the study type including whether data are quantitative, qualitative, or mixed-methods (e.g. qualitative cross-sectional, quantitative experimental, mixed-methods case study).*
Research sample	*State the research sample (e.g. Harvard university undergraduates, villagers in rural India) and provide relevant demographic information (e.g. age, sex) and indicate whether the sample is representative. Provide a rationale for the study sample chosen. For studies involving existing datasets, please describe the dataset and source.*

Sampling strategy	*Describe the sampling procedure (e.g. random, snowball, stratified, convenience). Describe the statistical methods that were used to predetermine sample size OR if no sample-size calculation was performed, describe how sample sizes were chosen and provide a rationale for why these sample sizes are sufficient. For qualitative data, please indicate whether data saturation was considered, and what criteria were used to decide that no further sampling was needed.*
Data collection	*Provide details about the data collection procedure, including the instruments or devices used to record the data (e.g. pen and paper, computer, eye tracker, video or audio equipment) whether anyone was present besides the participant(s) and the researcher, and whether the researcher was blind to experimental condition and/or the study hypothesis during data collection.*
Timing	*Indicate the start and stop dates of data collection. If there is a gap between collection periods, state the dates for each sample cohort.*
Data exclusions	*If no data were excluded from the analyses, state so OR if data were excluded, provide the exact number of exclusions and the rationale behind them, indicating whether exclusion criteria were pre-established.*
Non-participation	*State how many participants dropped out/declined participation and the reason(s) given OR provide response rate OR state that no participants dropped out/declined participation.*
Randomization	*If participants were not allocated into experimental groups, state so OR describe how participants were allocated to groups, and if allocation was not random, describe how covariates were controlled.*

Ecological, evolutionary & environmental sciences study design

All studies must disclose on these points even when the disclosure is negative.

Study description	*Briefly describe the study. For quantitative data include treatment factors and interactions, design structure (e.g. factorial, nested, hierarchical), nature and number of experimental units and replicates.*
Research sample	*Describe the research sample (e.g. a group of tagged Passer domesticus, all Stenocereus thurberi within Organ Pipe Cactus National Monument), and provide a rationale for the sample choice. When relevant, describe the organism taxa, source, sex, age range and any manipulations. State what population the sample is meant to represent when applicable. For studies involving existing datasets, describe the data and its source.*
Sampling strategy	*Note the sampling procedure. Describe the statistical methods that were used to predetermine sample size OR if no sample-size calculation was performed, describe how sample sizes were chosen and provide a rationale for why these sample sizes are sufficient.*
Data collection	*Describe the data collection procedure, including who recorded the data and how.*
Timing and spatial scale	*Indicate the start and stop dates of data collection, noting the frequency and periodicity of sampling and providing a rationale for these choices. If there is a gap between collection periods, state the dates for each sample cohort. Specify the spatial scale from which the data are taken*
Data exclusions	*If no data were excluded from the analyses, state so OR if data were excluded, describe the exclusions and the rationale behind them, indicating whether exclusion criteria were pre-established.*
Reproducibility	*Describe the measures taken to verify the reproducibility of experimental findings. For each experiment, note whether any attempts to repeat the experiment failed OR state that all attempts to repeat the experiment were successful.*
Randomization	*Describe how samples/organisms/participants were allocated into groups. If allocation was not random, describe how covariates were controlled. If this is not relevant to your study, explain why.*
Blinding	*Describe the extent of blinding used during data acquisition and analysis. If blinding was not possible, describe why OR explain why blinding was not relevant to your study.*

Did the study involve field work? ☐ Yes ☐ No

Field work, collection and transport

Field conditions	*Describe the study conditions for field work, providing relevant parameters (e.g. temperature, rainfall).*
Location	*State the location of the sampling or experiment, providing relevant parameters (e.g. latitude and longitude, elevation, water depth).*
Access and import/export	*Describe the efforts you have made to access habitats and to collect and import/export your samples in a responsible manner and in compliance with local, national and international laws, noting any permits that were obtained (give the name of the issuing authority, the date of issue, and any identifying information).*
Disturbance	*Describe any disturbance caused by the study and how it was minimized.*

Reporting for specific materials, systems and methods

Materials & experimental systems		
n/a	Involved in the study	
	☒	Unique biological materials
	☒	Antibodies
	☒	Eukaryotic cell lines
☒		Palaeontology
	☒	Animals and other organisms
	☒	Human research participants

Methods		
n/a	Involved in the study	
☒		ChIP-seq
☒		Flow cytometry
☒		MRI-based neuroimaging

Unique biological materials

Policy information about availability of materials

Obtaining unique materials: All unique materials used are readily available from the authors.

Antibodies

Antibodies used:
anti-m6A(Synaptic Systems, 202003, Rabbit)
anti-YTHDC1 (Abcam, ab122340, Rabbit);
anti-METTL3(Abcam, ab195352, Rabbit);
anti-IGF2BP2 (Proteintech, 11601-1-AP,Rabbit);
anti-Flag(Cell signaling Tech, 8146,Mouse);
anti-Myc(Cell signaling Tech, 2276,Mouse);
anti-NSUN2(Proteintech, 66580-1-Ig, Mouse);
anti-HMGA2 (Proteintech, 20795-1-AP, Rabbit);
anti-CXCR4 (Proteintech, 11073-2-AP, Rabbit);
anti-E-cadherin(BD Biosciences, 610405 Mouse);
anti-Vimentin(BD Biosciences, 550513, Mouse);
anti-GAPDH (Proteintech, 60004-1-Ig, Mouse; Proteintech, 10494-1-AP, Rabbit);
anti-IgG (Millipore, CS200621, Mouse; Millipore, PP64B, Rabbit);
Key Fluor 647 conjugated goat anti-rabbit IgG (H+L) (Keygen, KGAB015);

Validation: All the antibodies were validated with preliminary experiments. We examined primary antibodies according to manuals, and got similar results with validation results on manufacturer's website or relevant citations.

Eukaryotic cell lines

Policy information about cell lines

Cell line source(s): The DLD1 (ATCC CCL-221), HCT116 (ATCC CCL-247), LoVo (ATCC CCL-229), SW620 (ATCC CCL-227), SW1116 (ATCC CCL-233) and 293T (ATCC CRL-11268) cell lines, were purchased from American Type Culture Collection (ATCC). The TC71 patient-derived xenograft cell line was obtained from XENTECH (MRF reference: XTM-233_CXT-399/R5700).

Authentication: The HCT116 and DLD1 cell lines were authenticated by STR profiling

Mycoplasma contamination: All cell line tested negative for mycolasma contamination.

Commonly misidentified lines (See ICLAC register): The study did not involve commonly misidentified lines.

Palaeontology

Specimen provenance: *Provide provenance information for specimens and describe permits that were obtained for the work (including the name of the issuing authority, the date of issue, and any identifying information).*

Specimen deposition: *Indicate where the specimens have been deposited to permit free access by other researchers.*

Dating methods	*If new dates are provided, describe how they were obtained (e.g. collection, storage, sample pretreatment and measurement), where they were obtained (i.e. lab name), the calibration program and the protocol for quality assurance OR state that no new dates are provided.*

☐ Tick this box to confirm that the raw and calibrated dates are available in the paper or in Supplementary Information.

Animals and other organisms

Policy information about studies involving animals; ARRIVE guidelines recommended for reporting animal research

Laboratory animals	For liver metastasis model, 6 week-old female Balb/c nude mice were obtained from Vital River Laboratories (Beijing, China). For lung metastasis model, 4 week-old female Balb/c female mice were obtained from Vital River Laboratories (Beijing, China).
Wild animals	The study did not involve wild animals.
Field-collected samples	The study did not involve samples collected from the field.

Human research participants

Policy information about studies involving human research participants

Population characteristics	Human colorectal carcinoma (CRC) and adjacent normal tissues were collected from 97 patients receiving surgery with informed consent at Sun Yat-Sen University Cancer Center (SYSUCC) from 2005 to 2015. Clinical information of the CRC patients is summarized in Supplementary data 3. A total of 18 normal colorectal tissues and 22 colorectal adenomas were collected . In addition, 25 CRC tissues (including 20 pairs with matched liver metastasis (LM)) were obtained from SYSUCC. Clinical information of the CRC patients was summarized in Supplementary data 4. Serum samples from 18 healthy individuals, 20 CRC patients without LM and 20 CRC patients with LM were obtained from SYSUCC. Patient informed consent form was properly signed before recruitment for the study.
Recruitment	Participants were recruited from the pool of patients at SYSUCC. There may be self selection biases based on individuals who are able to seek care at these institutions. The sample should be representative of the populations served by the institutions.

ChIP-seq

Data deposition

☐ Confirm that both raw and final processed data have been deposited in a public database such as GEO.

☐ Confirm that you have deposited or provided access to graph files (e.g. BED files) for the called peaks.

Data access links<br>*May remain private before publication.*	*For "Initial submission" or "Revised version" documents, provide reviewer access links. For your "Final submission" document, provide a link to the deposited data.*
Files in database submission	*Provide a list of all files available in the database submission.*
Genome browser session<br>(e.g. UCSC)	*Provide a link to an anonymized genome browser session for "Initial submission" and "Revised version" documents only, to enable peer review. Write "no longer applicable" for "Final submission" documents.*

Methodology

Replicates	*Describe the experimental replicates, specifying number, type and replicate agreement.*
Sequencing depth	*Describe the sequencing depth for each experiment, providing the total number of reads, uniquely mapped reads, length of reads and whether they were paired- or single-end.*
Antibodies	*Describe the antibodies used for the ChIP-seq experiments; as applicable, provide supplier name, catalog number, clone name, and lot number.*
Peak calling parameters	*Specify the command line program and parameters used for read mapping and peak calling, including the ChIP, control and index files used.*
Data quality	*Describe the methods used to ensure data quality in full detail, including how many peaks are at FDR 5% and above 5-fold enrichment.*
Software	*Describe the software used to collect and analyze the ChIP-seq data. For custom code that has been deposited into a community repository, provide accession details.*

Flow Cytometry

Plots

Confirm that:

- [ ] The axis labels state the marker and fluorochrome used (e.g. CD4-FITC).
- [ ] The axis scales are clearly visible. Include numbers along axes only for bottom left plot of group (a 'group' is an analysis of identical markers).
- [ ] All plots are contour plots with outliers or pseudocolor plots.
- [ ] A numerical value for number of cells or percentage (with statistics) is provided.

Methodology

Sample preparation	*Describe the sample preparation, detailing the biological source of the cells and any tissue processing steps used.*
Instrument	*Identify the instrument used for data collection, specifying make and model number.*
Software	*Describe the software used to collect and analyze the flow cytometry data. For custom code that has been deposited into a community repository, provide accession details.*
Cell population abundance	*Describe the abundance of the relevant cell populations within post-sort fractions, providing details on the purity of the samples and how it was determined.*
Gating strategy	*Describe the gating strategy used for all relevant experiments, specifying the preliminary FSC/SSC gates of the starting cell population, indicating where boundaries between "positive" and "negative" staining cell populations are defined.*

- [ ] Tick this box to confirm that a figure exemplifying the gating strategy is provided in the Supplementary Information.

Magnetic resonance imaging

Experimental design

Design type	*Indicate task or resting state; event-related or block design.*
Design specifications	*Specify the number of blocks, trials or experimental units per session and/or subject, and specify the length of each trial or block (if trials are blocked) and interval between trials.*
Behavioral performance measures	*State number and/or type of variables recorded (e.g. correct button press, response time) and what statistics were used to establish that the subjects were performing the task as expected (e.g. mean, range, and/or standard deviation across subjects).*

Acquisition

Imaging type(s)	*Specify: functional, structural, diffusion, perfusion.*
Field strength	*Specify in Tesla*
Sequence & imaging parameters	*Specify the pulse sequence type (gradient echo, spin echo, etc.), imaging type (EPI, spiral, etc.), field of view, matrix size, slice thickness, orientation and TE/TR/flip angle.*
Area of acquisition	*State whether a whole brain scan was used OR define the area of acquisition, describing how the region was determined.*

Diffusion MRI ☐ Used ☐ Not used

Preprocessing

Preprocessing software	*Provide detail on software version and revision number and on specific parameters (model/functions, brain extraction, segmentation, smoothing kernel size, etc.).*
Normalization	*If data were normalized/standardized, describe the approach(es): specify linear or non-linear and define image types used for transformation OR indicate that data were not normalized and explain rationale for lack of normalization.*
Normalization template	*Describe the template used for normalization/transformation, specifying subject space or group standardized space (e.g. original Talairach, MNI305, ICBM152) OR indicate that the data were not normalized.*
Noise and artifact removal	*Describe your procedure(s) for artifact and structured noise removal, specifying motion parameters, tissue signals and physiological signals (heart rate, respiration).*
Volume censoring	*Define your software and/or method and criteria for volume censoring, and state the extent of such censoring.*

Statistical modeling & inference

Model type and settings	*Specify type (mass univariate, multivariate, RSA, predictive, etc.) and describe essential details of the model at the first and second levels (e.g. fixed, random or mixed effects; drift or auto-correlation).*
Effect(s) tested	*Define precise effect in terms of the task or stimulus conditions instead of psychological concepts and indicate whether ANOVA or factorial designs were used.*

Specify type of analysis: ☐ Whole brain ☐ ROI-based ☐ Both

Statistic type for inference (See Eklund et al. 2016)	*Specify voxel-wise or cluster-wise and report all relevant parameters for cluster-wise methods.*
Correction	*Describe the type of correction and how it is obtained for multiple comparisons (e.g. FWE, FDR, permutation or Monte Carlo).*

Models & analysis

n/a	Involved in the study
☐	☐ Functional and/or effective connectivity
☐	☐ Graph analysis
☐	☐ Multivariate modeling or predictive analysis

Functional and/or effective connectivity	*Report the measures of dependence used and the model details (e.g. Pearson correlation, partial correlation, mutual information).*
Graph analysis	*Report the dependent variable and connectivity measure, specifying weighted graph or binarized graph, subject- or group-level, and the global and/or node summaries used (e.g. clustering coefficient, efficiency, etc.).*
Multivariate modeling and predictive analysis	*Specify independent variables, features extraction and dimension reduction, model, training and evaluation metrics.*
