# Supplementary material for: N6-methyladenosine modification of circNSUN2 facilitates cytoplasmic export and stabilizes HMGA2 to promote colorectal liver metastasis
Source: Nat Commun. 2019 Oct 16;10:4695. doi: 10.1038/s41467-019-12651-2 (PMC6795808; doi:10.1038/s41467-019-12651-2)
Supplement: Supplementary file 3 — Description of Additional Supplementary Files [file 41467_2019_12651_MOESM3_ESM.doc]

**Description of Additional Supplementary Files**

File Name: Supplementary Data 1

Description: Candidates of potential regulatory circRNAs in CRCs

File Name: Supplementary Data 2

Description: Primer, siRNA and shRNA sequences

File Name: Supplementary Data 3

Description: Patient Information of SYSUCC CRC Cohort for qPCR analysis

File Name: Supplementary Data 4

Description: Patient Information of SYSUCC CRC Cohort with liver metastasis for qPCR analysis
